# Influence of the gut microbiota on the pharmacokinetics of tacrolimus in liver transplant recipients: insights from microbiome analysis

**DOI:** 10.3389/fmicb.2025.1616985

**Published:** 2025-09-22

**Authors:** Yan Wang, Zhongyuan Bai, Yufeng Liu, Yan Wang, Jun Xu, Zhiyong Lai

**Affiliations:** ^1^First Clinical Medical College, Shanxi Medical University, Taiyuan, China; ^2^Microbiological Laboratory of Ophthalmology, Shanxi Eye Hospital, Taiyuan, China; ^3^Department of Hepatobiliary Surgery and Liver Transplantation Center, First Hospital of Shanxi Medical University, Taiyuan, China; ^4^Shanxi Key Laboratory of Digestive Diseases and Organ Transplantation, First Hospital of Shanxi Medical University, Taiyuan, China; ^5^Department of Biliopancreatic Surgery, First Hospital of Shanxi Medical University, Taiyuan, China

**Keywords:** gut microbiome, tacrolimus, pharmacokinetics, liver transplantation, ATP-binding cassette transporters, immunosuppressive therapy

## Abstract

**Introduction:**

Tacrolimus is crucial for immunosuppression after liver transplantation, but its pharmacokinetics vary markedly among individuals. Emerging evidence suggests that the gut microbiota may influence its metabolism, although the underlying mechanisms remain unclear.

**Methods:**

This study analyzed the fecal microbiota from 38 postliver transplant patients and 31 healthy controls via 16S rDNA amplicon and shotgun metagenomic sequencing. Patients were stratified into three groups on the basis of oral tacrolimus dosage and blood concentration: LDLBC (low dose, low blood concentration), LDHBC (low dose, high blood concentration), and SDLBC (standard dose, low blood concentration).

**Results:**

Posttransplant patients presented significantly reduced gut microbial diversity. Specific bacterial taxa, including *Enterococcus raffinosus*, *Intestinibacter bartlettii*, and *Bacteroides fragilis*, were enriched in patients with lower tacrolimus blood concentrations. In contrast, *Phascolarctobacterium faecium* and *Streptococcus salivarius* were associated with increased drug levels. Functional analysis revealed differences between patient subgroups in ATP-binding cassette (ABC) transporters and drug efflux pumps, suggesting a potential microbial influence on tacrolimus absorption and metabolism. Additionally, antibiotic resistance genes were more abundant in patients with lower tacrolimus blood concentrations, particularly in the *Escherichia coli*-enriched groups.

**Discussion:**

These findings underscore the influence of the gut microbiota on tacrolimus pharmacokinetics and support the potential of microbial composition as a biomarker for optimizing immunosuppressive therapy.

## 1 Introduction

Liver transplantation is an effective treatment for end-stage liver disease, yet rejection remains an independent risk factor for long-term survival (Kosuta et al., 2024). Immunosuppressive drugs effectively prevent and treat rejection, and most liver transplant centers in China currently favor a multidrug regimen based on calcineurin inhibitors. Tacrolimus is the most commonly used calcineurin inhibitor. Following oral administration, tacrolimus is primarily absorbed in the small intestine (Mohammed Ali et al., 2023) and metabolized in the liver by cytochrome P450 enzymes (CYPs), mainly CYP3A4 and CYP3A5 (Tillman et al., 2023). Additionally, the efflux pump transporter ATP-binding cassette subfamily B member 1 (ABCB1) in the cell membrane can transport tacrolimus back into the intestinal lumen (Henkel et al., 2023). Owing to its narrow therapeutic window, complex drug interactions, and poor correlation between dose and blood concentration, tacrolimus dosing requirements, efficacy, and toxicity vary significantly among patients (Campagne et al., 2019). Consequently, physicians routinely monitor tacrolimus blood concentrations, although individual variability may result in subtherapeutic or supratherapeutic concentrations that can lead to rejection or transplant complications (Tremblay et al., 2017; Campagne et al., 2019). The most crucial cause of interindividual variability is the difference in CYP3A5 genotype (Csikany et al., 2021). However, most liver transplant centers do not routinely test CYP3A5 genotypes to guide immunosuppressant therapy.

Recent research on the gut microbiome suggests that microbial diversity may contribute to interindividual variability in tacrolimus pharmacokinetics and pharmacodynamics (Guo et al., 2019). Immunosuppressants after liver transplantation can also alter the intestinal microecology (Sucu et al., 2023). Disturbance of the intestinal microecology can lead to the release of inflammatory factors, liver function damage, and graft dysfunction and even correlate with increased mortality after transplantation (Swarte et al., 2022). Some studies have shown that *Subdoligranulum* is associated with a significantly reduced risk of death after liver transplantation, whereas *Enterococcus* is linked to an increased risk of all-cause mortality (Choudhary et al., 2017).

Few studies have examined the relationship between immunosuppressants and intestinal microecology in solid organ transplantation, and most remain at the animal research stage (Jiang et al., 2018; Degraeve et al., 2023). Thus, the underlying mechanism requires further elucidation. For example, an increased tacrolimus dose during the first month after kidney transplantation is positively correlated with the abundance of *Faecalibacterium prausnitzii* in the first postoperative week (Lee et al., 2015). *In vitro* experiments have subsequently shown that most *Clostridiales bacteria* can metabolize tacrolimus (Guo et al., 2019). In our study, we combined 16S rDNA amplicon sequencing with shotgun metagenomics sequencing to further analyze the changes in the intestinal flora after liver transplantation. We also investigated the correlations between the gut microbiota and clinical characteristics.

## 2 Materials and methods

### 2.1 Study design

**Figure 1:**
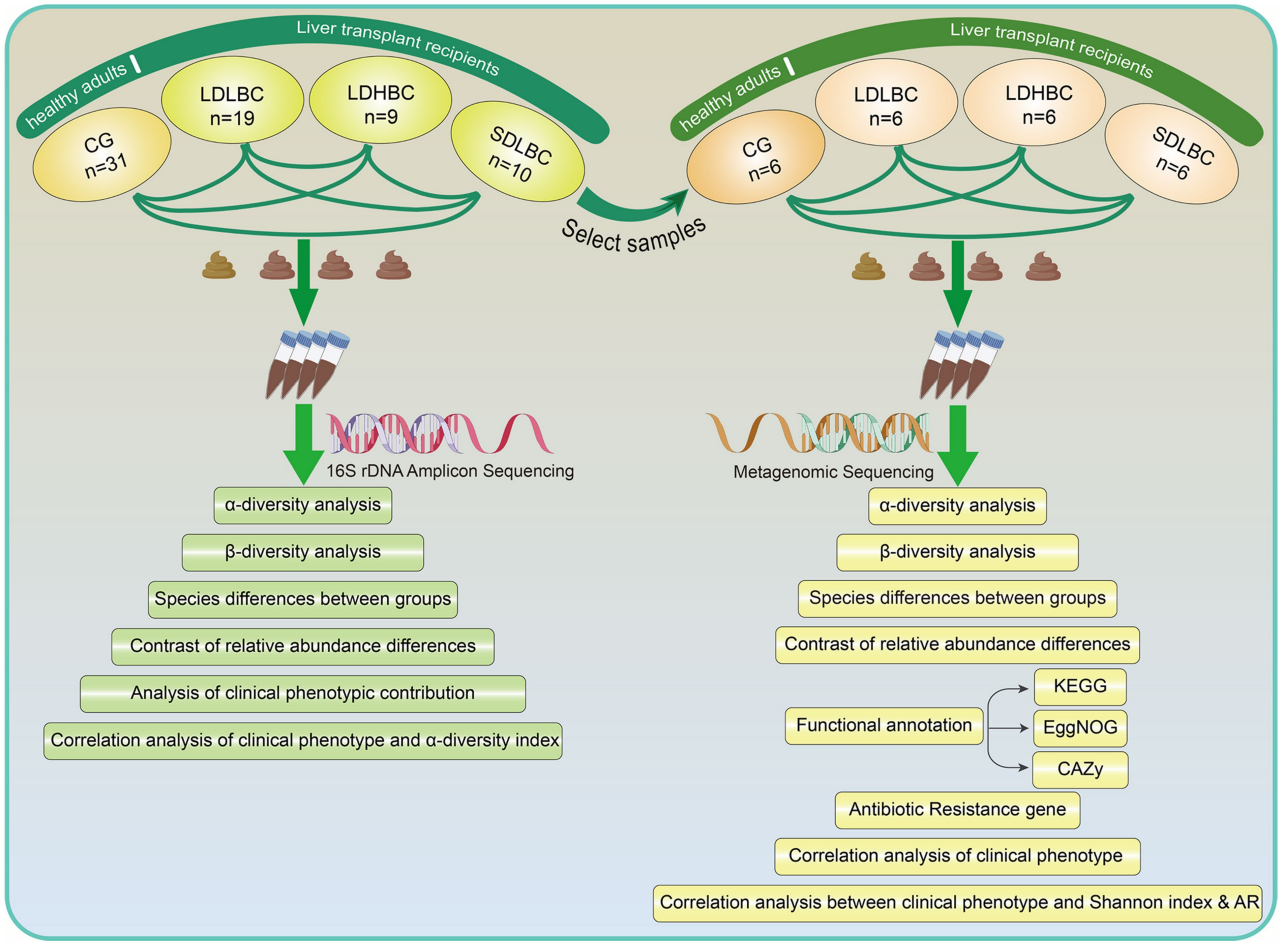
Research methods and processes.

We prospectively collected fecal samples from 38 patients who underwent liver transplantation at the First Hospital of Shanxi Medical University from 2021 to 2023. In addition, 31 healthy adults were recruited as the control group (CG). We classified the 38 postliver transplantation patients into three groups according to Chinese immunosuppressive therapy guidelines (Transplantation, 2021): the LDLBC group (low dose, low blood concentration, *n* = 19), the LDHBC group (low dose, high blood concentration, *n* = 9) and the SDLBC group (Standard dose, low blood concentration, *n* = 10). According to the guidelines (Transplantation, 2021), a tacrolimus dose of 0.05–0.15 mg·kg^−1^·d^−1^ is recommended for oral combination therapy in two divided doses. None of the patients included in this study exceeded the recommended oral dosage range. We classified patients with oral tacrolimus doses below the recommended range into the low-dose group, whereas the other patients were categorized into the standard-dose group. The target blood concentration ranges of tacrolimus are 8–12 ng/mL during the first 3 months posttransplant, 7–10 ng/mL between 3 and 6 months, 6–8 ng/mL between 6 and 12 months, and approximately 5 ng/mL after 12 months. For the postoperative period, we defined patients with blood tacrolimus concentrations below the reference range as the low blood concentration group and those with concentrations equal to or above the lower limit of the reference range as the high blood concentration group. Clinical data, including medical history, laboratory test results, clinical manifestations, and disease classifications, were obtained from medical records and the laboratory information system. We first performed 16S rDNA amplicon sequencing analysis, followed by the discriminant method in microPITA software (v1.0.1) to select six representative samples from the center of each group (Supplementary Figure S1A). These selected samples were then further subjected to shotgun metagenomic sequencing (Figure 1). In addition, we conducted an association analysis between clinical characteristics and the gut microbiota and developed a predictive model to assess the impact of clinical features and the gut microbiota on tacrolimus intrapatient variability (Tac-IPV).

The detailed methods for fecal sample collection, 16S rDNA amplicon sequencing, metagenomic sequencing, and bioinformatics analysis employed in this study are provided in the online Supplementary materials and methods.

### 2.2 Statistical analysis

Clinical data were analyzed via chi-square tests or one-way ANOVA to assess intergroup differences, with multiple comparisons performed via Tukey’s multiple comparison tests or the Kruskal–Wallis test. Statistical analyses were conducted via QIIME2 (v202202), LEfSe (v1.1.01), Perl (v5.26.2), and R software (v4.0.3). Feature selection was performed via the random forest and SHAP methods. Model prediction analysis was performed via a combination of cross-validation, bootstrap, random forest, and extreme gradient boosting (XGBoost) methods. A *p* value < 0.05 was considered statistically significant.

## 3 Results

### 3.1 Patient information

Our grouping is justified by the differences in tacrolimus dosage (TACD) and blood drug concentration (BCD) within the study group (Table 1). The healthy group exhibited a significantly lower average age than the study groups did (*p* < 0.05). Except for preoperative aspartate aminotransferase (AST) (LDHBC vs. LDLBC, *p* < 0.05), no significant differences were observed in the collected baseline clinical data (Table 1; Supplementary Table S1). Notably, the LDLBC group had the highest proportion of patients with liver malignancies, whereas the SDLBC group had the greatest proportion of patients with ascites. Additionally, the LDHBC group presented no complications such as hypertension, diabetes, or malignancies, and these differences may be worth our consideration and further exploration. We introduced the Tac-IPV index to better elucidate the variability between the tacrolimus oral dose and blood concentration. We quantified this variability via the coefficient of variation (CV), a widely adopted metric (Xie et al., 2024). The results demonstrated that the CV in the LDHBC group was significantly lower than that in the LDLBC group (*p* < 0.05).

**Table 1:** Cohort characteristics.

Clinical characteristics	SDLBC	LDHBC	LDLBC	CG	*p*-value
	(*N* = 10)	(*N* = 9)	(*N* = 19)	(*N* = 31)	
General characteristics					
Gender (male)	6 (60.00%)	6 (66.70%)	15 (78.90%)	18 (58.10%)	0.4924
Age (years)	56.3 (9.63)^a^	51.44 (10.69)^a^	53.63 (8.83)^a^	40.1 (9.29)	<0.0001
BMI (kg/m^2^)	22.35 (3.42)	21.52 (2.3)	23.71 (3.23)	22.28 (2.36)	0.1926
Complications					
AIT (yes)	1 (10.00%)	3 (33.30%)	4 (21.10%)	–	0.4603
DM (yes)	4 (40.00%)	0	5 (26.30%)	–	0.1143
HTN (yes)	2 (20.00%)	0	4 (21.10%)	–	0.3301
Cancer (yes)	1 (10.00%)	0^b^	9 (47.40%)	–	0.0115
Ascites (yes)	6 (60.00%)^b^	2 (22.20%)	3 (15.80%)	–	0.039
EF%	64.5 (3.63)	64.11 (6.1)	67.21 (4.77)	–	0.1973
Preoperative lab tests					
HB (g/L)	111.1 (16.97)	107 (28.27)	127.3 (24.5)	–	0.0934
WBC (*10^9^/L)	3.12 (0.87)	3.96 (1.98)	4.33 (2.2)	–	0.2754
TBIL (μmol/L)	96.01 (139.65)	106.02 (90.27)	139.36 (201.72)	–	0.8371
ALT (U/L)	31.43 (16.29)	55.5 (82.84)	49.57 (36.24)	–	0.0993
AST (U/L)	47.9 (22.13)	35.44 (39.39)^b^	51.89 (24.42)	–	0.0298
GGT (U/L)	75.5 (93.06)	45 (46.97)	73.37 (71.39)	–	0.3192
ALP (U/L)	448.5 (927)	96.11 (61.22)	253.4 (400.4)	–	0.0559
ALB (g/L)	33.5 (18.4)	37.82 (6.4)	36.21 (5.16)	–	0.6598
TC (mmol/L)	3.5 (2.19)	2.9 (1.63)	3.41 (1.45)	–	0.7046
LDL (mmol/L)	2.36 (1.13)	1.72 (0.59)	2.24 (0.95)	–	0.291
HDL (mmol/L)	1.13 (0.49)	1.08 (0.44)	1.04 (0.33)	–	0.8464
Sampling lab tests					
SALT (U/L)	22.2 (15.5)	12.22 (3.38)	19.11 (13.4)	–	0.0574
SAST (U/L)	21 (11.2)	16.44 (4.8)	23.79 (15.06)	–	0.2929
STBIL (μmol/L)	13.6 (5.12)	16.76 (10.28)	16.31 (9.31)	–	0.787
TIME (month)	9.5 (7.43)	11 (7.92)	8.316 (5.218)	–	0.5954
TACD (mg/d)	4.1 (1.1)^bc^	2.44 (0.92)	1.78 (0.85)	–	<0.0001
BCD (ng/ml)	4.39 (1.88)^c^	7.92 (1.18)^b^	3.58 (2.08)	–	<0.0001
Tac-IPV (CV)	43.31 (42.45)	20.09 (15.16)^b^	37.26 (32.71)	–	0.0312

The data were analyzed via chi-square tests or ANOVA for group differences, with Tukey’s test or Kruskal–Wallis test for multiple comparisons. Data are n (%) or means (SD). BMI, Body Mass Index; DM, Diabetes Mellitus; HTN, Hypertension; Cancer, Liver Cancer; EF%, Ejection Fraction; HB, Hemoglobin; WBC, White Blood Cell; ALT, Alanine Aminotransferase; AST, Aspartate Aminotransferase; ALB, Albumin; TBIL, Total Bilirubin; GGT, Gamma-Glutamyl Transferase; ALP, Alkaline Phosphatase; TC, Total Cholesterol; LDL, Low-Density Lipoprotein; HDL, High-Density Lipoprotein; AIT, Anti-infective therapy; SALT, Sampling Alanine Aminotransferase; SAST, Sampling Aspartate Aminotransferase; STBIL, Sampling Total Bilirubin; TIME, Postoperative duration; TACD, Tacrolimus Dosage; BCD, Blood drug concentrations; Tac-IPV (CV), Tacrolimus Intrapatient variability (Coefficient of Variation).

a *P* < 0.05* versus the CG group.

b *P* < 0.05* versus the LDLBC group.

c *P* < 0.05* versus the LDHBC group.

### 3.2 Analysis of the species community composition

**Figure 2:**
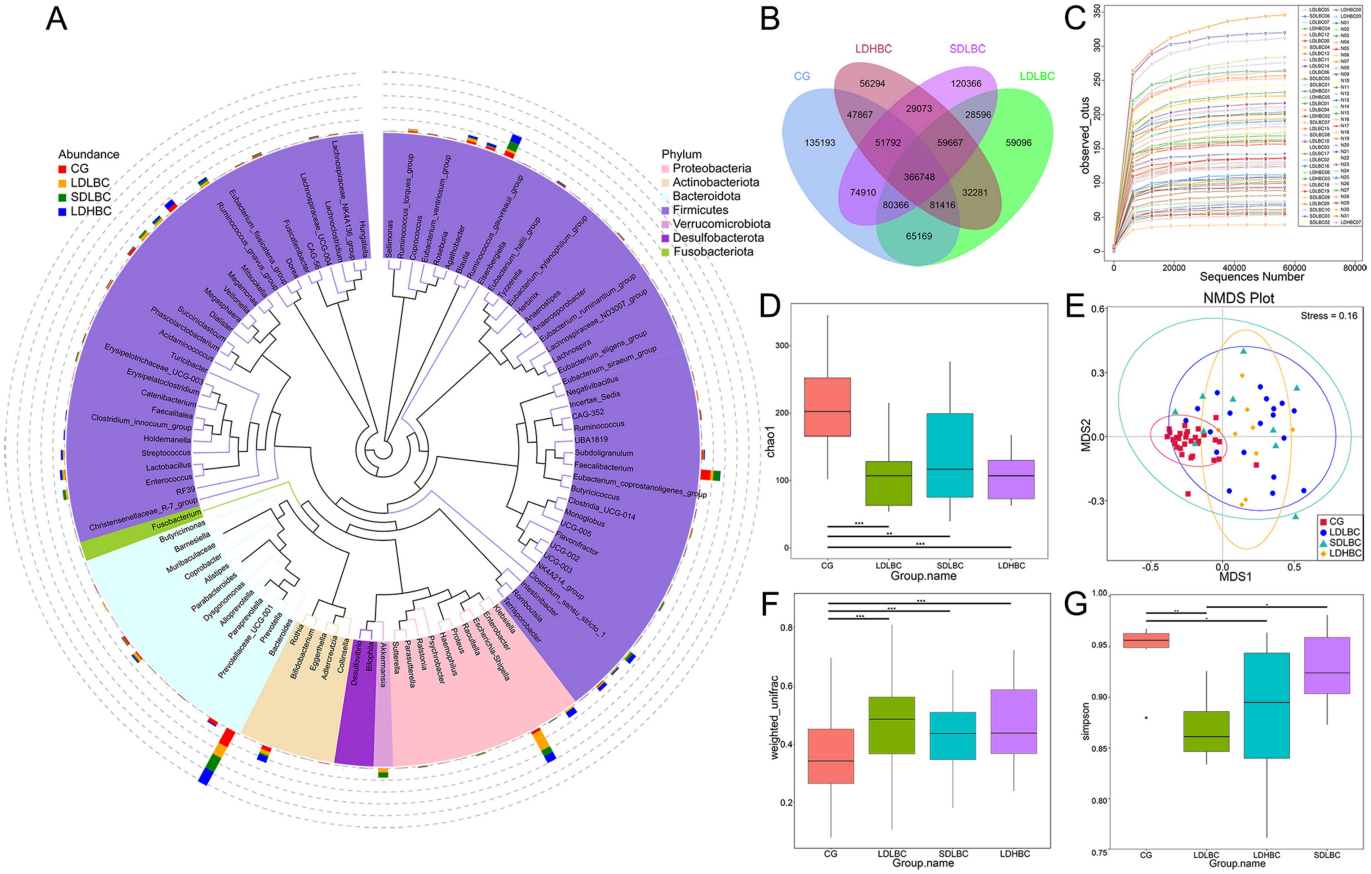
Changes in the intestinal microbiota structure after liver transplantation. **(A)** Phylogenetic tree of the top 100 genera based on 16S rDNA amplicon sequencing. The colors of the sectors represent phyla, and the stacked bar charts on the outer rings indicate bacterial genus abundance across groups. The left legend denotes group information, whereas the legend shows phylum level classification. **(B)** Venn diagram of the metagenomics sequence data. **(C)** Differences in the observed feature indices across all samples. **(D)** Differences in the Chao1 index between the four groups based on 16S rDNA amplicon sequencing. **(E)** NMDS analysis of weighted Unifrac distances between the four groups on the basis of 16S rDNA amplicon sequencing. Each point represents a sample; distances between points reflect differences in community structure (Stress < 0.2 indicates reliable NMDS analysis). **(F)** Beta–Wilcox weighted Unifrac differences between the four groups were determined via 16S rDNA amplicon sequencing. **(G)** Differences in the Simpson index at the species level between groups, based on metagenomic sequence data (*p < 0.05, **p < 0.01, ***p < 0.001).

In our research, compared with the 84 amplicon sequence variants unannotated via 16S rDNA amplicon sequencing, 87,033 gene catalogs (unigenes) were unannotated via metagenomic sequencing. Overall, The top five phyla identified via 16S rDNA amplicon and metagenomic sequencing accounted for 99.80 and 88.78% of the total annotated microorganisms, respectively (Figure 2A; Supplementary Table S2). Metagenomic sequencing characterizes the shared and unique gene information among different groups. The CG group had the most specific gene sequences, followed by the SDLBC group, and the LDLBC and LDHBC groups had almost equal numbers of specific gene sequences (Figure 2B).

### 3.3 Changes in the intestinal microbial structure after liver transplantation

We first used 16S amplicon sequencing to characterize the diversity of the intestinal flora in patients after liver transplantation. The observed feature indices indicated that the sequencing depth of the 16S rDNA amplicon sequencing was reasonable (Figure 2C). The species diversity of the liver transplantation groups (LDLBC, LDHBC, and SDLBC groups) was significantly lower than that of the CG group (*p* < 0.001) (Figure 2D). The results of deep metagenomic sequencing were also intuitive (GENEBOX diagram) (Supplementary Figure S1B). Differences in the abundance and evenness of the gut microbiota between liver transplant recipients and healthy individuals may be related to intestinal congestion caused by preoperative cirrhosis and portal hypertension (Wu et al., 2023), brief anhepatic periods during surgery (Lai et al., 2022), and perioperative antibiotic use (Fu et al., 2023). The nonmetric multidimensional scaling (NMDS) results also support the uniqueness of the CG group, and the spatial distance difference among the three groups after liver transplantation was relatively small (Figures 2E,F). In the metagenomic sequencing results, the Simpson index was significantly different among the LDHBC, LDLBC, and CG groups (*p* < 0.05). There were significant differences between the LDLBC and SDLBC groups (*p* < 0.05), suggesting that the LDLBC group is unique and that its dominant species may not have a high abundance (Figure 2G). To further verify the uniqueness of the LDLBC group, we performed NMDS analysis on the metagenomic sequencing results. The results at the phylum, class, order, family, genus, and species levels were consistent with those of 16S rDNA amplicon sequencing (all with stress < 0.2) (Supplementary Figure S1C). The CG group presented the greatest distance from the other groups at each classification level, followed by the LDLBC group.

### 3.4 Analysis of species differences between groups

**Figure 3:**
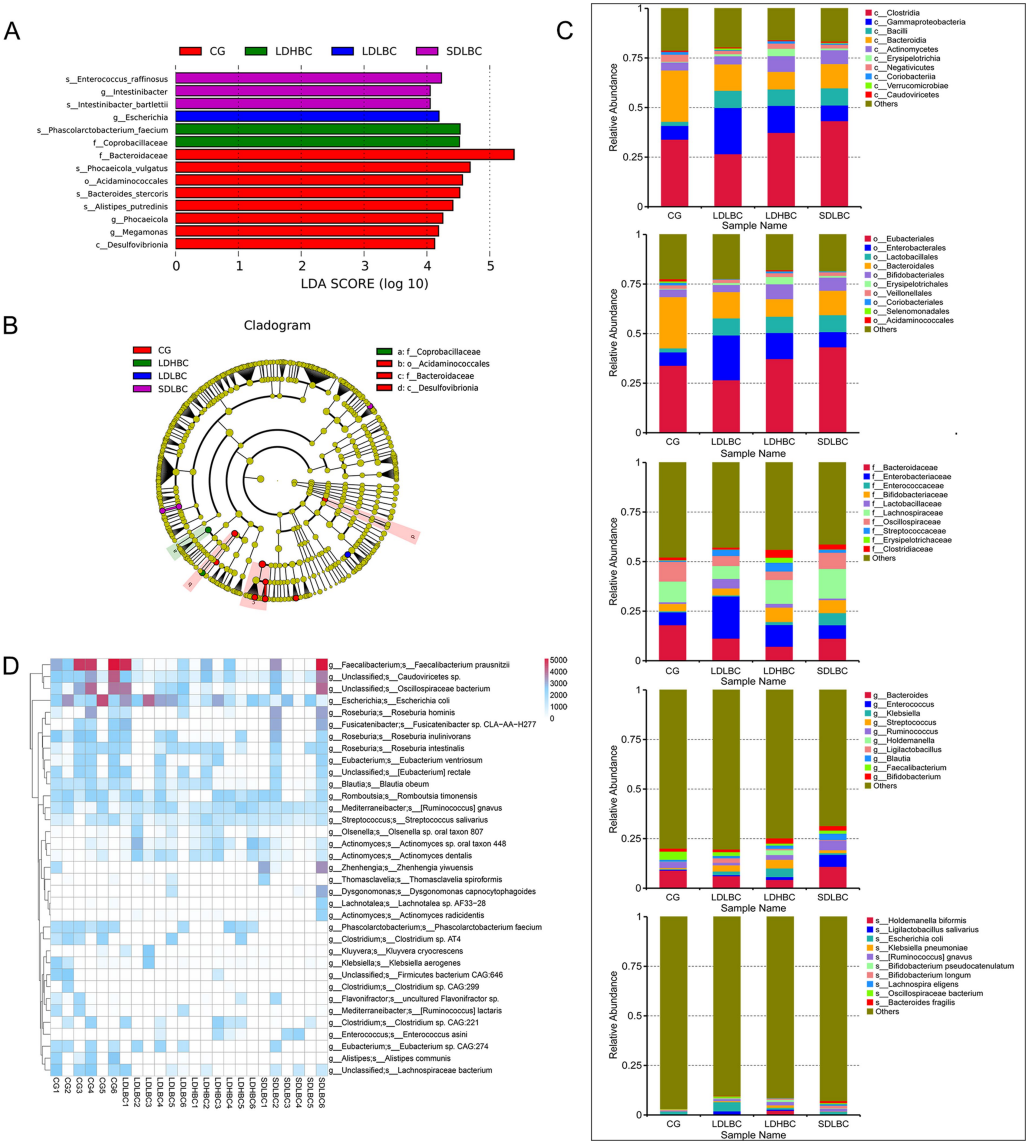
Species differences between groups annotated by metagenomic sequencing. **(A)** Characteristic bacteria were identified between groups via LEfSe analysis on the basis of metagenomic sequence data (LDA > 4). **(B)** Clade diagram of species that differ between groups. Circles represent taxonomic levels from phylum to species, with circle size indicating abundance. Yellow circles represent species with no significant differences, whereas colored circles indicate distinct species biomarkers. **(C)** Bar plot showing the abundances of the top 10 species by abundance at each taxonomic level (class, order, family, genus, and species) on the basis of the metagenomic sequencing data. **(D)** Heatmap of gene abundance differences between samples at the species level, based on GENENUMS analysis.

Linear discriminant analysis effect size (LEfSe) analysis (Linear Discriminant Analysis, LDA > 4) based on metagenomic sequencing revealed statistically significant differences among the CG, LDLBC, LDHBC, and SDLBC groups (Figures 3A,B; Supplementary Table S3). In the SDLBC group, I*ntestinibacter*, *Intestinibacter bartlettii*, and *Enterococcus raffinosus* were dominant. The abundance of *Phascolarctobacterium faecium* was significantly increased in the LDHBC group (LDA = 4.53, *p* < 0.05). *Phascolarctobacterium faecium* promotes digestion, nutrient absorption, immune regulation, and antibacterial activity and maintains the gut microbiota balance and host health (Wu et al., 2017; Ikeyama et al., 2020). Furthermore, comparisons between the LDLBC and LDHBC groups revealed that *Streptococcus* and *Streptococcus sali*var*ius* were more abundant in the LDHBC group (LDA = 4.59, *p* < 0.05) (Supplementary Figure S2B; Supplementary Table S3). Similarly, 16S rDNA amplicon sequencing also revealed a significant increase in the abundances of *Streptococcaceae* and *Streptococcus* in the LDHBC group. In the LDLBC group, *Escherichia* differed significantly from that in the other groups (LDA = 4.19, *p* < 0.05). Moreover, subgroup analysis revealed that both *Escherichia* and *Escherichia coli* were more abundant in the LDLBC group than in the LDHBC (Supplementary Figure S2B; Supplementary Table S3) and SDLBC groups (Supplementary Figure S2C; Supplementary Table S3). 16S rDNA amplicon sequencing revealed that the LDLBC group presented statistically significant differences in the abundances of *Proteobacteria*, *Gammaproteobacteria*, *Enterobacterales*, *Enterobacteriaceae*, and *Escherichia Shigella* (the old NCBI hierarchical classification; Supplementary Figure S2A; Supplementary Table S4). These successive taxonomic units showed significant differences, indicating that the observed variations originated at lower classification levels. Similarly, the CG group demonstrated significant differences across taxonomic ranks from high to low levels. For example, *Bacteroidaceae, Phocaeicola, Bacteroides stercoris, Phocaeicola vulgatus*, and *Alistipes putredinis* were significantly different (Figure 3A; Supplementary Table S3).

### 3.5 Relative abundance differences observed between groups

We considered that differences in the relative abundance of species could better reflect intergroup species differences when a small sample size was used. We performed the analysis primarily on the basis of the metagenomic sequencing results (Figure 3C), using 16S rDNA amplicon sequencing as a reference. At the class level, the relative abundance of *Bacteroidia* was greater in the CG group than in all the other three groups (the genera *Bacteroides stercoris*, *Phocaeicola vulgatus*, and *Alistipes putredinis* were all dominant in the CG group). Our results are consistent with those of previous studies, which revealed that the abundance of *Bacteroidia* in cirrhotic mice was lower than that in healthy mice (Chen R. et al., 2024). *Bacilli* (containing *Streptococcus salivarius* predominant in the LDHBC group, and *Enterococcus raffinosus* predominant in the SDLBC), as potential probiotics, were relatively low in the CG group and relatively high in the other three groups, which may be closely related to the use of oral probiotic preparations during the postoperative recovery period (Tang et al., 2024). The most abundant bacterial class in the LDLBC group was *Gammaproteobacteria* (with the subordinate taxa *Enterobacteriaceae* and *Escherichia coli* being dominant in LDLBC). *Erysipelotrichia* was the most abundant class in the LDHBC group (with the subordinate taxon *Coprobacillaceae* being dominant in LDHBC). Consistent with the class level, at the order level, the relative abundance of *Bacteroidales* was greater in the CG group than in the other three groups; the relative abundance of *Lactobacillales* was low in the CG group but high in the other three groups; *Enterobacterales* was the greatest in the LDLBC group; and *Erysipelotrichales* was the most abundant in the LDHBC group. At the family level, we observed that *Enterobacteriaceae* had the highest relative abundance in the LDLBC group. Interestingly, at the order level, the *Lactobacillales* with high relative abundance in the three groups subjected to liver transplantation presented different high-abundance microbial taxa in their subordinate families: the relative abundance of *Lactobacillaceae* was the highest in the LDLBC group; the relative abundance of *Enterococcaceae* was the highest in the SDLBC group; compared with the other groups, *Streptococcaceae* was more abundant in the LDHBC group. These flora may play different roles in the absorption or metabolism of tacrolimus. Consistent with the 16S rDNA amplicon sequencing results (Supplementary Figure S2D), the metagenomic sequencing results at the genus level revealed that *Faecalibacterium* was highly abundant in the CG group (Figure 3C). Although the difference in the relative abundance of species level bacteria gradually decreased, *Escherichia coli* still had a high relative abundance in the LDLBC group. *Escherichia coli* is an opportunistic pathogen that can ferment various sugars to produce acid and gas, which is usually not conducive to the postoperative recovery of patients (Han et al., 2024). We also obtained species level sequencing results consistent with the 16S rDNA amplicon sequencing results (Supplementary Figure S2D), and *Bacteroides fragilis* was significantly more abundant in the SDLBC group (Figure 3C). *Bacteroides fragilis* has beneficial functions such as immune regulation. However, it may also become an opportunistic pathogen under specific conditions, which deserves attention (Chen Z. et al., 2024).

The heatmap results revealed that the relative abundance of *Faecalibacterium prausnitzii* was significantly greater in the CG group than in the other groups (Supplementary Figure S1D; Supplementary Table S5). In terms of gene counts at the species level, *Faecalibacterium prausnitzii* was present almost exclusively in the CG group and at relatively high counts (Figure 3D). Our results differ from those of a previous study, which revealed that patients requiring increased oral tacrolimus doses had higher abundances of *Faecalibacterium prausnitzii* in their gut microbiota. This discrepancy may be due to our smaller sample size; another possibility is that the previous study was conducted within 1 month postoperatively when the patients’ conditions were not entirely stable and their digestive function had not fully recovered.

### 3.6 Functional annotation

#### 3.6.1 The CAZy database

From the perspective of gene annotations at various levels, the microbial carbohydrate metabolism enzymes of liver transplant recipients were significantly lower than those of healthy individuals (Supplementary Figure S3). This result may be due to the decreased abundance of the intestinal microbiota after liver transplantation. These enzymes belong mainly to glycosyl transferase family 2 (GT2) and glycosyl transferase family 4 (GT4) (Supplementary Figure S3A). Both are involved in catalyzing the transfer of glycosyl groups from one compound to another, thereby participating in protein glycosylation and the synthesis and degradation of microbial cell walls or the extracellular matrix. The synthesis and modification of biomolecules are essential for microorganism survival, reproduction, and environmental adaptation (Bretagne et al., 2024) (Supplementary Figure S3B). These enzymes are indispensable for maintaining normal physiological function, cell communication, the immune response, and other critical processes in living organisms.

#### 3.6.2 The EggNOG database

The EggNOG database is an essential tool and database for functional annotation and gene family analysis (Supplementary Figure S4A). The functional annotation at the OG level (ortholog group ID) revealed that the LDHBC group annotated a greater number of COG1136 (ATP-binding cassette transporter (ABC transporter); lipoprotein transporter activity) and COG1131 (ABC transporter; ATPase activity) functional groups (Supplementary Figure S4B; Supplementary Table S6). The SDLBC group included COG1132 (ABC transporter; ATPase activity, coupled to transmembrane movement of substances) and ENOG501TP0B (ABC transporter) (Supplementary Figure S4B). These protein groups are all related to drug transport (Montanari and Ecker, 2015). However, at the same time, the SDLBC group presented relatively high abundances of the COG0577 (efflux transmembrane transporter activity) and COG0534 (Mate efflux family protein; drug transmembrane transporter activity) functional groups (Supplementary Figure S4B), which may be one of the reasons for the low serum concentration of tacrolimus (Demel et al., 2008). We also found that histidine kinase activity was relatively low in the LDLBC group and high in the SDLBC group (Supplementary Figures S4B,C). Previous studies have shown that *Lactobacillus* can inhibit the growth of pathogens by producing bacteriocins (Younas et al., 2022). Bacteriocin production depends on the induction peptide (IP) acting on histidine protein kinase (HPK) to activate the network regulation of cytoplasmic response regulator (RR), a two-component regulatory system. Previous studies have shown that acetate promotes bacteriocin synthesis in *Lactobacillus plantarum* (Meng et al., 2021a). Acetate activates *Lactobacillus plantarum, Lactobacillus sakei*, and *Lactobacillus rhamnosus* to produce plantaricin E, plantaricin F, sakacin A, and rhamnosin B by activating histidine kinase activity (Meng et al., 2021b). Despite the higher abundance of Lactobacillus in the LDLBC group and the higher histidine kinase activity in the SDLBC group, both groups may have produced fewer bacteriocins, which remains to be further verified. It is also possible that the lower production of bacteriocins in the LDLBC and SDLBC groups was related to the lower serum tacrolimus concentrations.

#### 3.6.3 The KEGG database

**Figure 4:**
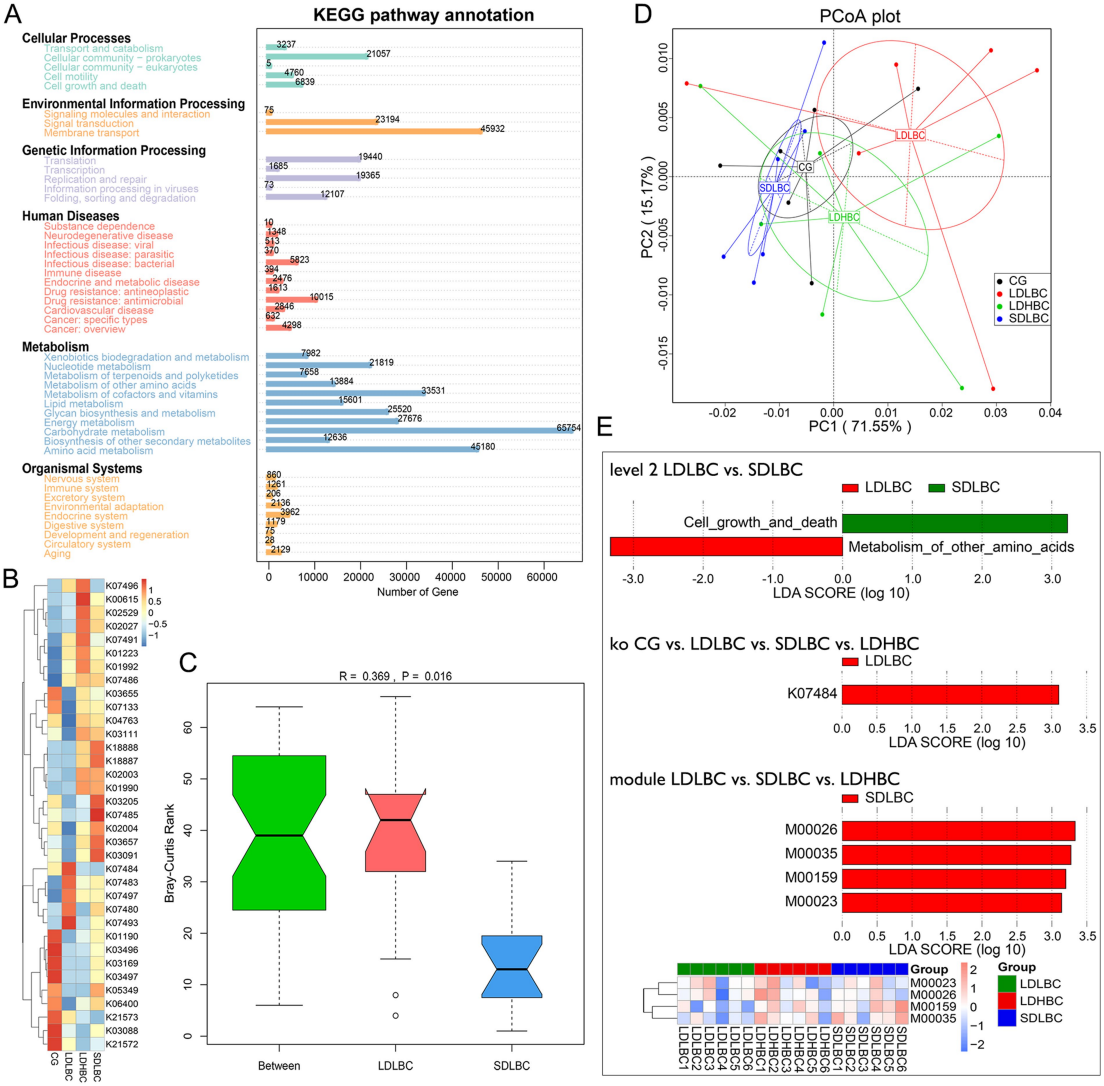
Function Comments on the basis of the KEGG database. **(A)** Numbers of annotated genes in the KEGG database (six function classes). **(B)** Cluster analysis of functional relative abundance at the KEGG ortholog (KO) level. **(C)** Anosim was used to compare the functional abundance between the two groups at the module level (KEGG module number). An R value > 0 indicates that between-group differences are more significant than within-group differencesare, whereas an R value < 0 suggests the opposite. A *p* value < 0.05 indicates statistical significance. **(D)** Dimensionality reduction analysis of Bray–Curtis distance (PCoA) on the basis of module level functional abundance. **(E)** LEfSe analysis of functional differences based on the KEGG database (LDA > 3) (Level 2: LDLBC vs. SDLBC; KO: CG vs. LDLBC vs. SDLBC vs. LDHBC; Module: LDLBC vs. SDLBC vs. LDHBC; includes an abundance clustering heatmap for significantly different functions).

Our functional annotation results revealed that more than half of the genes were annotated to the metabolism pathway (Figure 4A). The Environmental Information Processing, Cellular Processes, Human Diseases, and Metabolism functional groups were relatively abundant in the LDLBC group (Supplementary Figure S5A; Supplementary Table S7). Metastats analysis revealed that 13 level 2 functions significantly differed between the CG and LDLBC groups (Supplementary Figure S6A; Supplementary Table S8). The more accurate classification of functional notes suggests that relatively high functions in the LDLBC group include drug resistance, antimicrobial, infectious disease, bacterial, and cellular community-prokaryote functions (Supplementary Figure S6B). Cellular community-prokaryotes were annotated as biofilm formation - *Escherichia coli* at Level 3 (Supplementary Figure S5B; Supplementary Table S7), possibly due to the predominance of *Escherichia coli* in the LDLBC group. The SDLBC group presented greater abundances of K02003 (putative ABC transport system ATP-binding protein), K01990 (ABC-2 type transport system ATP-binding protein), and K02004 (putative ABC transport system permease protein) within the KEGG ortholog group (KO) (Figure 4B; Supplementary Table S7). Furthermore, K18888 and K18887 (ATP-binding cassette, subfamily B, multidrug efflux pump) were also significantly enriched within the SDLBC group (Figure 4B). We also found that K01992 (ABC-2 type transport system permease protein) was highly abundant in the LDHBC group (Figure 4B). Consistent with the annotation results of the CAZy database, the protein groups responsible for transport function were all low in abundance in the LDLBC group, which may explain why an appropriate dose of oral tacrolimus resulted in lower blood concentrations. However, the specific mechanism underlying the high abundance of this multidrug efflux pump is unknown. In addition, the relative abundances of transposase and putative transposase were significantly greater in the three liver transplant groups but lower in the CG group (Figure 4B), suggesting that microbial gene expression regulation is active in the guts of liver transplant recipients. Our analysis of functional abundance at the module level revealed that the intergroup difference between the LDLBC and SDLBC groups was more significant than the intragroup difference. Moreover, principal co-ordinates analysis (PCoA) based on the Bray–Curtis distance revealed that the distance between the SDLBC and LDLBC groups was the greatest (Figures 4C,D). To further clarify the functional differences between groups, we performed LEfSe analysis on the functional annotations at each level (Figure 4E; Supplementary Table S9). The LEfSe analysis (LDA > 3) results indicated that the findings were broadly consistent with those reported above. Compared with those in the LDLBC and SDLBC groups, cell growth and death were significantly higher in the SDLBC group (LDA = 3.23, *p* < 0.05), and the metabolism of other amino acids was significantly greater in the LDLBC group (LDA = 3.33, *p* < 0.05). The differences in CG vs. LDLBC vs. LDHBC vs. SDLBC at the KO level revealed that the LDLBC group presented a greater abundance of K07484 transposase (LDA = 3.10, *p* < 0.05). This result was consistent with the greater abundance of the LDLBC group COG2963 (transposase activity) function group, whose genes were annotated in the EggNOG database. At the module level, through comparative analysis of the differences between LDLBC vs. LDHBC vs. SDLBC, we found that the different functions of the SDLBC group were M00026 (histidine biosynthesis, 5-phospho-alpha-D-ribose 1-diphosphate → histidine), M00035 (methionine degradation), M00159 (V/A-type ATPase, prokaryotes), and M00023 (tryptophan biosynthesis, chorismite → tryptophan).

Another significant finding from the KEGG database annotation was pathwaymaps (Supplementary Pathwaymaps). By comparing different groups, we observed that the gut microbes in the LDHBC group play a diminished role in the pathway promoting steroid production. Previous studies have shown that glucocorticoids induce CYP3A4 and ABCB1 in drug interactions, leading to a significantly higher tacrolimus dose requirement after kidney transplantation when glucocorticoids are coadministered (Anglicheau et al., 2003). The locus of enterocyte effacement encoded effectors, which are specific to the LDLBC group and play a vital role in intestinal pathogens such as enteropathogenic *Escherichia coli* (EPEC) and enterohemorrhagic *Escherichia coli* (EHEC). Prior reports have indicated increased intestinal *Escherichia coli* after liver and kidney transplantation and urinary tract colonization by pathogenic *Escherichia coli* (Swarte et al., 2022). After liver transplantation, the proliferation of *Escherichia coli* in patients’ intestines leads to the production of abundant virulence effector proteins, ultimately causing diarrhea (Kaur and Dudeja, 2023). Moreover, enteritis caused by infectious diarrhea can lead to a short-term increase in tacrolimus blood concentrations (Nakamura et al., 2014). Additionally, we found that the Ras homolog gene family, member A (RhoA), was expressed explicitly in multiple metabolic pathways in the LDHBC group, playing crucial roles in cell signal transduction and cytoskeletal recombination. In contrast, Son of Sevenless (SOS), which promotes the conversion of the Ras protein from its inactive state (RAS GDP) to its active state (RAS GTP) and is closely linked to various clinical diseases (Bandaru et al., 2019), was not detected in the SDLBC group. Multiple signaling pathways in the LDLBC and LDHBC groups revealed explicit SOS expression in cell proliferation, signal transduction, and immune function pathways. Previous studies have confirmed that SOS promotes cell proliferation. SOS1 can activate the Ras protein, and the Ras protein mutation is a critical factor in various tumors (Luo et al., 2023). However, the reason for the absence of SOS in the intestinal microbial metabolic pathway of the SDLBC group remains unclear.

### 3.7 Antibiotic resistance gene

**Figure 5:**
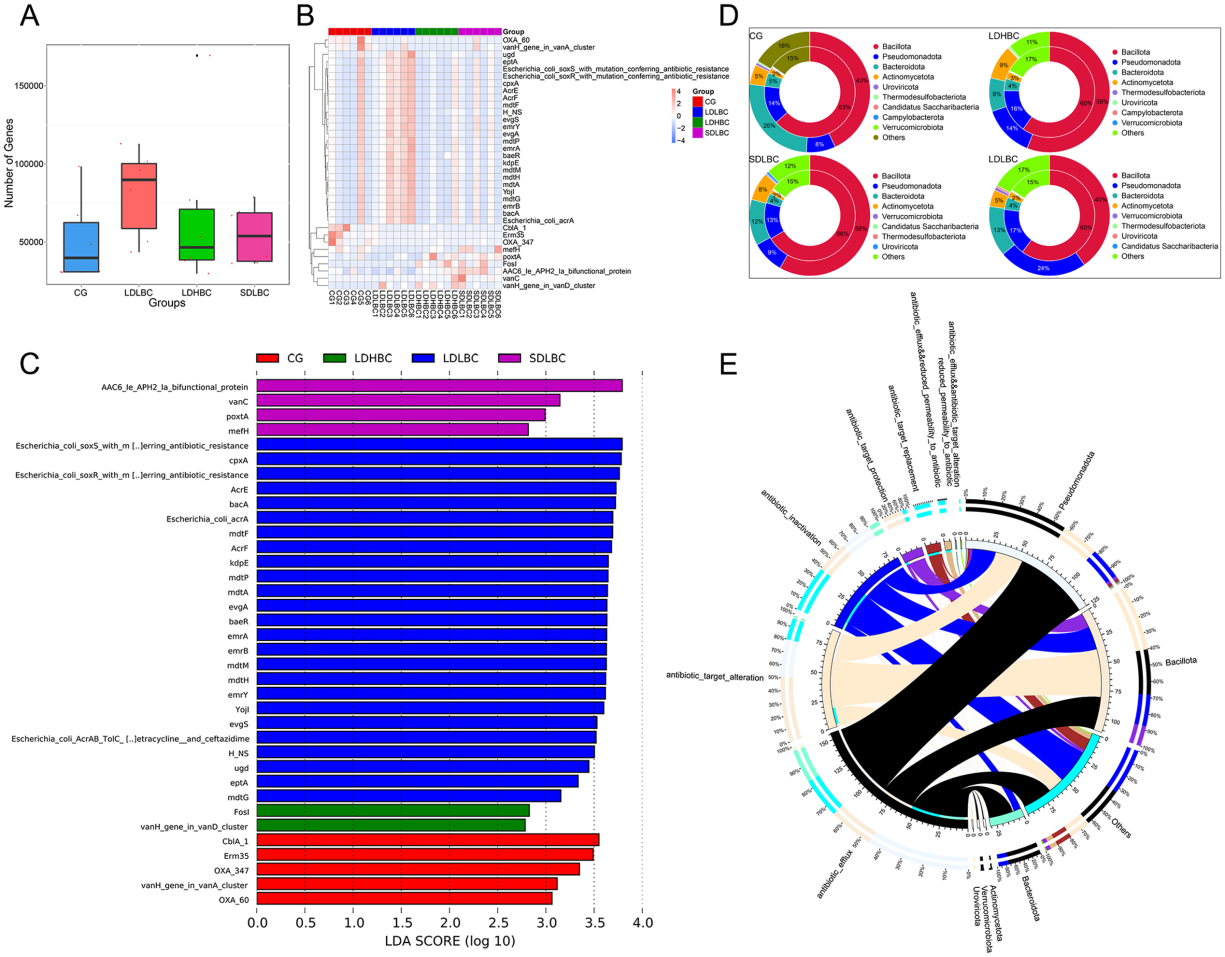
Resistance gene annotation on the basis of the CARD database. **(A)** Box plot showing the number of resistance genes across groups. **(B,C)** LEfSe analysis of resistance gene differences (LDA > 2); above is a clustering heatmap of the abundance of differential resistance genes between groups. **(D)** Two-circle map of the relationships between resistance genes and phylum level flora. The inner circle depicts the species distribution of ARO, whereas the outer circle illustrates the species distribution of genes across all samples, directly reflecting the enrichment of resistance genes within specific species. **(E)** Overview-circle diagram of resistance mechanisms and species (the right side depicts information on phylum level species, and the left side shows data on resistance mechanisms. The inner ring color indicates the resistance mechanism corresponding to each species).

Our results indicated that the LDLBC group had the most antibiotic resistance (AR) genes (Figure 5A), including several mutant resistance genes in *Escherichia coli* (Figures 5B,C; Supplementary Table S10), which may be the reason for the high abundance of *Escherichia coli* in that group. Association analysis of resistance genes and species affiliation revealed that *Bacillota, Bacteroidota*, and *Pseudomonadota* were the top three categories. These three categories accounted for more than 80% of each group’s annotation of resistance genes. *Pseudomonadota* had the most annotated resistance genes within the LDLBC group compared to other groups. The group with the most resistance genes corresponding to *Bacillota* was the SDLBC group, and the SDLBC group had the most *Bacillota* (Figure 5D). Studies have shown that Bacillus in probiotic preparations has extensive antibiotic resistance (Jin et al., 2024), suggesting that patients in the SDLBC group may be at potential risk of AR and bacterial pathogenicity. The overview-circle diagram (Figure 5E) further corroborated these findings.

### 3.8 Correlation analysis of clinical characteristics

**Figure 6:**
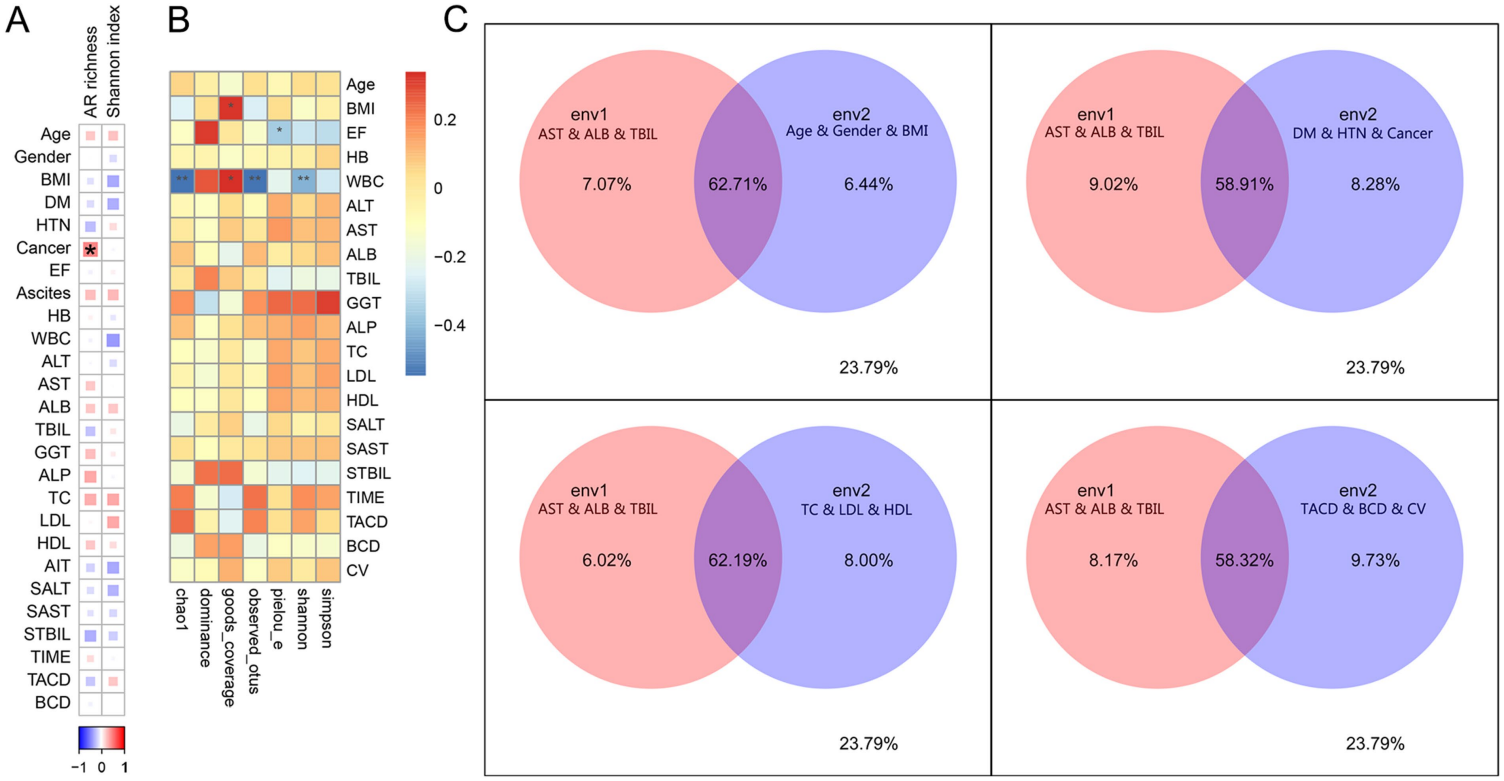
Correlation analysis of clinical characteristics. **(A)** Spearman correlation analysis between clinical characteristics and the Shannon index (species) and AR genes, on the basis of metagenomic sequence data. **(B)** Spearman correlation analysis between clinical characteristics and the α diversity index, based on 16S rDNA amplicon sequencing data. **(C)** Contribution of clinical characteristics to intestinal microbiota ecology (*p < 0.05, **p < 0.01).

We performed Spearman correlation analysis between the collected clinical characteristics and the metagenomic sequencing annotations of the gut microbes. The results revealed that *Fusobacteriales* were significantly positively correlated with T. RATE (TACD/BCD) (*p* < 0.001) and significantly negatively correlated with B. RATE (BCD/TACD) (*p* < 0.001) (Supplementary Figure S7A), suggesting that *Fusobacteriales* are abundant in the intestines of patients who have taken more tacrolimus orally and whose blood concentrations are low. We found that the WBC count was positively correlated with several common pathogenic bacteria (*p* < 0.05), such as *Klebsiella* and *Escherichia*; TBIL was positively correlated with *Ligilactobacillus sali*var*ius* and *Streptococcus sali*var*ius* (*p* < 0.05). STBIL and SAST were positively correlated with *Erysipelotrichales* (*p* < 0.05) and negatively correlated with *Klebsiella* and *Flavonifractor plautii* (*p* < 0.05) (Supplementary Figures S7A–C). In addition, we found a significant correlation between liver cancer and AR richness (*p* < 0.05) (Figure 6A), which was consistent with previous scholars’ findings. AR occurs in patients with tumors due to neutropenia caused by prophylactic or therapeutic antibiotics or chemotherapy (Nanayakkara et al., 2021). We also found that ALP and TC were positively correlated with AR richness: that the preoperative lipid indices (TC and LDL) were positively correlated with the Shannon index; and that WBC, AIT, BMI, and DM were negatively correlated with the Shannon index (Figure 6A). However, none of these correlations reached statistical significance. In addition, species richness and evenness were significantly correlated with some clinical phenotypes, as revealed by 16S amplicon sequencing annotation analysis (Figure 6B). In conclusion, these clinical characteristics influence the community distribution of the intestinal microecology in patients after liver transplantation. The general characteristics of liver transplant patients (age, gender, BMI) and underlying conditions (DM, HTN, cancer) explained less variance in the gut microbiome distribution than did preoperative liver function parameters (AST, ALB, TBIL). In contrast, preoperative lipid profiles (TC, LDL, HDL) and tacrolimus-related parameters (TACD, BCD, CV) had greater explanatory power for microbiome distribution (Figure 6C).

### 3.9 Prediction model

**Figure 7:**
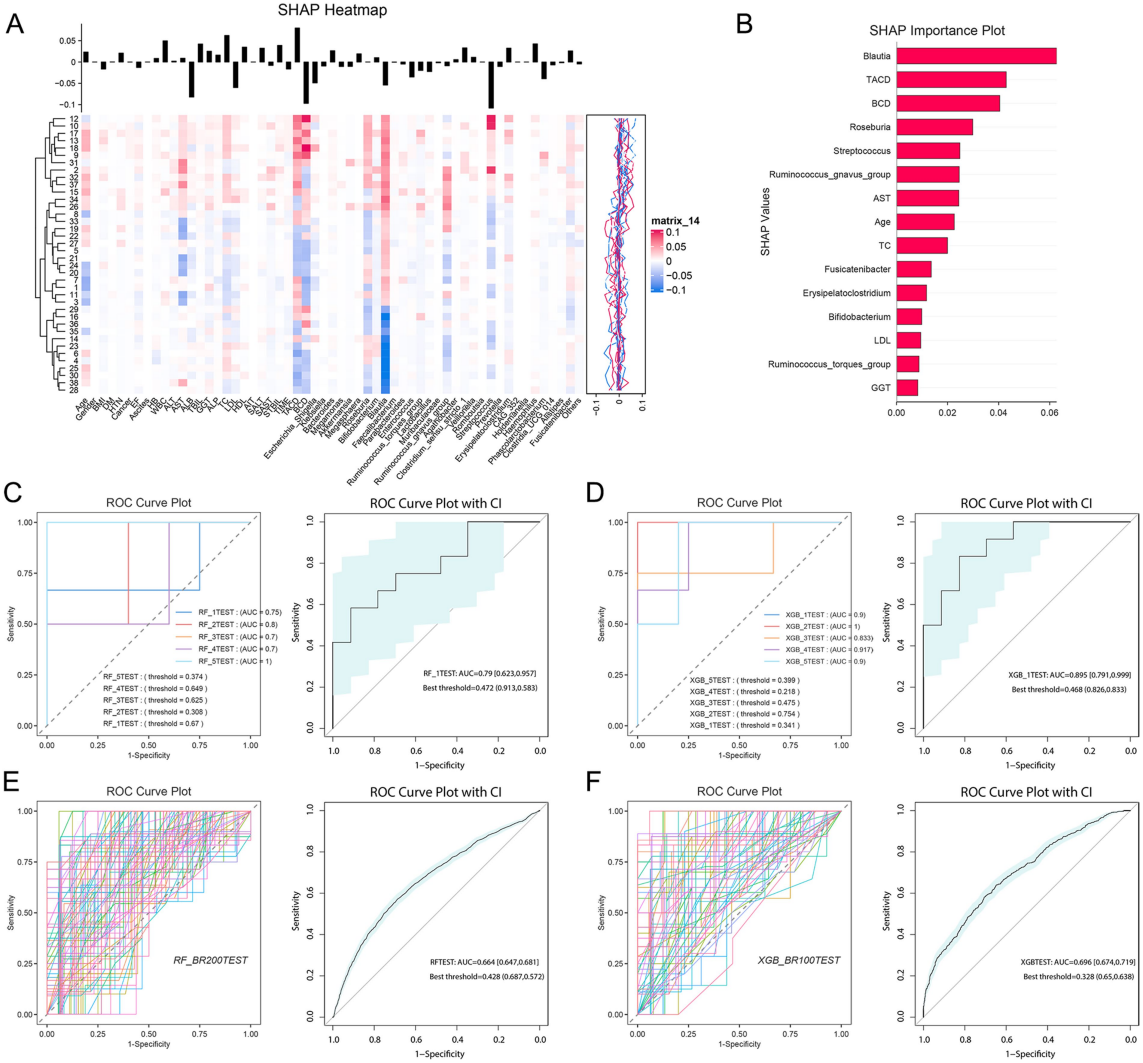
Model prediction analysis of tacrolimus intrapatient variability (coefficient of variation) on the basis of representative clinical characteristics and top flora. **(A)** SHAP heatmap illustrating the correlation between 26 clinical features, the top 30 flora, and the coefficient of variation. The vertical axis represents patient IDs, and the horizontal axis denotes feature names. The black vertical line above each feature indicates the distribution range of its corresponding SHAP values. **(B)** SHAP bar chart displaying the importance ranking of feature variables affecting tacrolimus intrapatient variability. **(C)** ROC curve plot of cross-validation results using random forest. **(D)** ROC curve plot of the cross-validation results obtained via XGBoost. **(E)** ROC curve plot of the bootstrap results obtained via random forest. **(F)** ROC curve plot of the bootstrap results obtained via XGBoost.

We stratified 38 patients by tacrolimus CV into high CV (CV > 30, *n* = 13) and low CV (CV < 30, *n* = 25) groups (Xie et al., 2024). A predictive model was constructed with a high CV as the outcome, incorporating clinical features and the top 30 abundant genera. Top eight variables (*Blautia*, TACD, BCD, *Roseburia*, *Streptococcus*, *Ruminococcus gnavus group*, AST, and Age) were selected (Figures 7A,B; Supplementary Table S11). The results indicated that clinical features, including Streptococcus, effectively predict tacrolimus variability in liver transplant patients (Figures 7C–F; Supplementary Table S12). Notably, *Blautia, Roseburia*, and *Ruminococcus gnavus* belong to the *Lachnospiraceae* family, while *Streptococcus* was enriched in the LDHBC group and may be linked to increased tacrolimus blood concentrations. Additionally, previous studies have reported that tacrolimus use is associated with reduced *Lachnospiraceae* (Han et al., 2019).

## 4 Discussion

In this study, we observed alterations in the intestinal microecological structure of postliver transplantation patients compared with healthy individuals. *Bacilli* dominated the intestinal microbiota of liver transplant recipients. Group comparisons revealed that patients with low oral doses and high blood concentrations exhibited a predominance of *Phascolarctobacterium faecium* and *Streptococcus salivarius* in the gut. Research has identified *Streptococcus salivarius* as a pathogenic bacterial group associated with various diseases (Gacesa et al., 2022). *Enterococcus raffinosus, Intestinibacter bartlettii*, and *Bacteroides fragilis* predominate in the intestines of patients with appropriate oral doses of tacrolimus and blood concentrations below the recommended values. Studies have shown that *Enterococcus raffinosus* and *Bacteroides fragilis* can usually cause pathogenic infections (Lee et al., 2022; Patrick, 2022). The increase in these harmful bacteria may hinder the absorption of tacrolimus or promote its metabolism. Our results suggested that patients with low-dose tacrolimus and low blood concentrations had a greater abundance of *Escherichia coli* in the gut, including associated AR and protein functional groups. Although previous studies have documented this phenomenon (Kaur and Dudeja, 2023; Han et al., 2024), the mechanism by which *Escherichia coli* influences tacrolimus pharmacokinetics remains unclear. We also observed differences in the functional groups of the ABC transporter and drug efflux pump, which may directly cause differences in the oral dose and blood concentration. However, these findings are preliminary, and due to potential heterogeneity, further validation through *in vitro* or animal experiments is required.

Although our results confirmed that tacrolimus pharmacokinetics are related to intestinal microecology, they do not resolve the issue of causality. Multiple factors contribute to changes in the intestinal microecology after liver transplantation. In addition to recipient liver disease and transplantation procedures (Lai et al., 2022; Wu et al., 2023), various immunosuppressants play a key role in altering the gut microbiota. Postoperative immunosuppressive therapy impairs immune function and damages the intestinal immune barrier, leading to imbalances in the gut microbiota (Xu and Zhang, 2015; Tourret et al., 2017). Previous studies have shown that moderate doses of tacrolimus increase the abundance of *Faecalibacterium prausnitzii* and *Bifidobacterium* in the intestinal tract of rats after liver transplantation (Jiang et al., 2018). Furthermore, mouse experiments have shown that mycophenolate mofetil can selectively induce bacterial expression of β-glucuronidase (Taylor et al., 2019). Conversely, enzymes produced by intestinal microorganisms directly influence the transformation and metabolism of various drugs, including tacrolimus and mycophenolate mofetil (Zimmermann et al., 2019).

This study has several limitations. First, we did not include preoperative fecal samples from liver transplant patients. Although this cross-sectional study explored the relationships among tacrolimus oral dosage, blood concentration, and the gut microbiota composition, it did not fully account for the potential influence of preexisting liver disease and surgical intervention on the gut microbial landscape. Second, the relatively small sample size and group imbalance may have introduced bias and limited the generalizability of our findings. In future studies, a longitudinal design incorporating pre- and posttransplant samples from the same individuals would provide more robust evidence and clearer insights into the effects of transplantation and immunosuppression on the gut microbiome. Additionally, the study did not include CYP3A5 genotyping for liver transplant patients, which may have contributed to an overinterpretation of the causal role of gut microbiota in tacrolimus metabolism. To address these limitations, our research team has initiated follow-up studies incorporating genetic profiling, which will be published as soon as relevant findings are available.

In conclusion, our study revealed that the diversity and abundance of gut microbes in patients after liver transplantation are lower than those in healthy adults. *Enterococcus raffinosus, Intestinibacter bartlettii*, *Bacteroides fragilis*, and *Fusobacteriales* in the intestinal microbiota of postliver transplant recipients may be associated with decreased tacrolimus blood concentrations. *Phascolarctobacterium faecium* and *Streptococcus salivarius* may be associated with high blood concentrations of tacrolimus. The ABC transport enzyme ensures the absorption of tacrolimus as much as possible, but the drug efflux pump protein leads to difficulty in maintaining the blood drug concentration. Although there are many findings in our study, the small sample size may lead to errors in the results, and it is still necessary to expand the sample size or conduct *in vitro* or animal experiments for verification.

## Data Availability

The original contributions presented in the study are publicly available. This data can be found at: https://www.ncbi.nlm.nih.gov/, PRJNA1200201.
