# Supplementary material for: Influence of the gut microbiota on the pharmacokinetics of tacrolimus in liver transplant recipients: insights from microbiome analysis
Source: Front Microbiol. 2025 Sep 22;16:1616985. doi: 10.3389/fmicb.2025.1616985 (PMC12498155; doi:10.3389/fmicb.2025.1616985)
Supplement: Supplementary file 1 [file Supplementary_file_1.zip › Supplementary Figure Legends.DOCX]

**Supplementary Figure Legends**


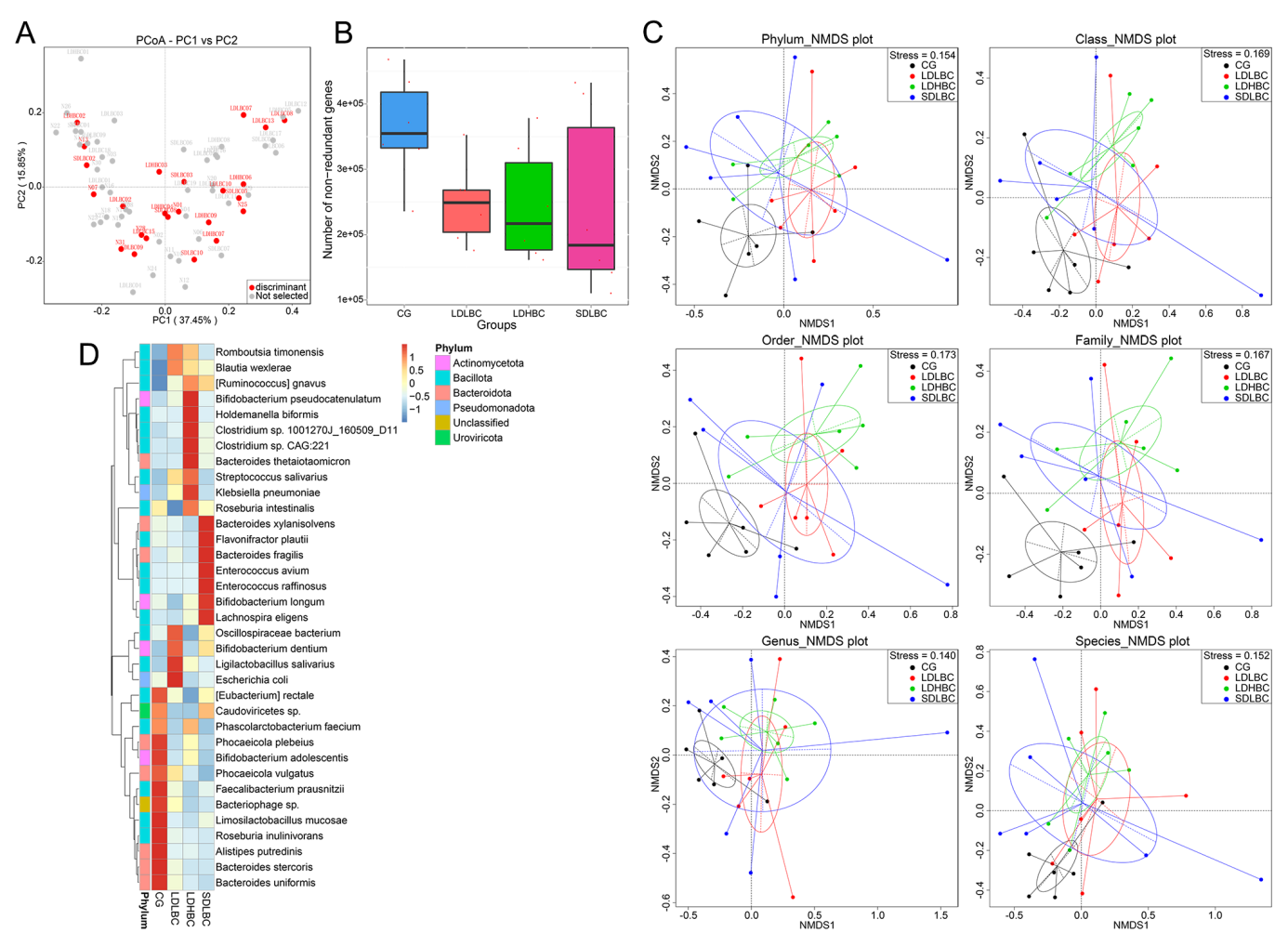


**Figure S1. Species diversity based on metagenomic sequencing.** **(A)** The discriminant method was used to select six representative samples from the center of each group for metagenomic sequencing. **(B)** GENEBOX plot based on the number of genes identified by metagenomic sequences. **(C)** NMDS analysis of species at each taxonomic level on the basis of metagenomic sequences. **(D)** Species level relative abundance clustering heatmap.

**
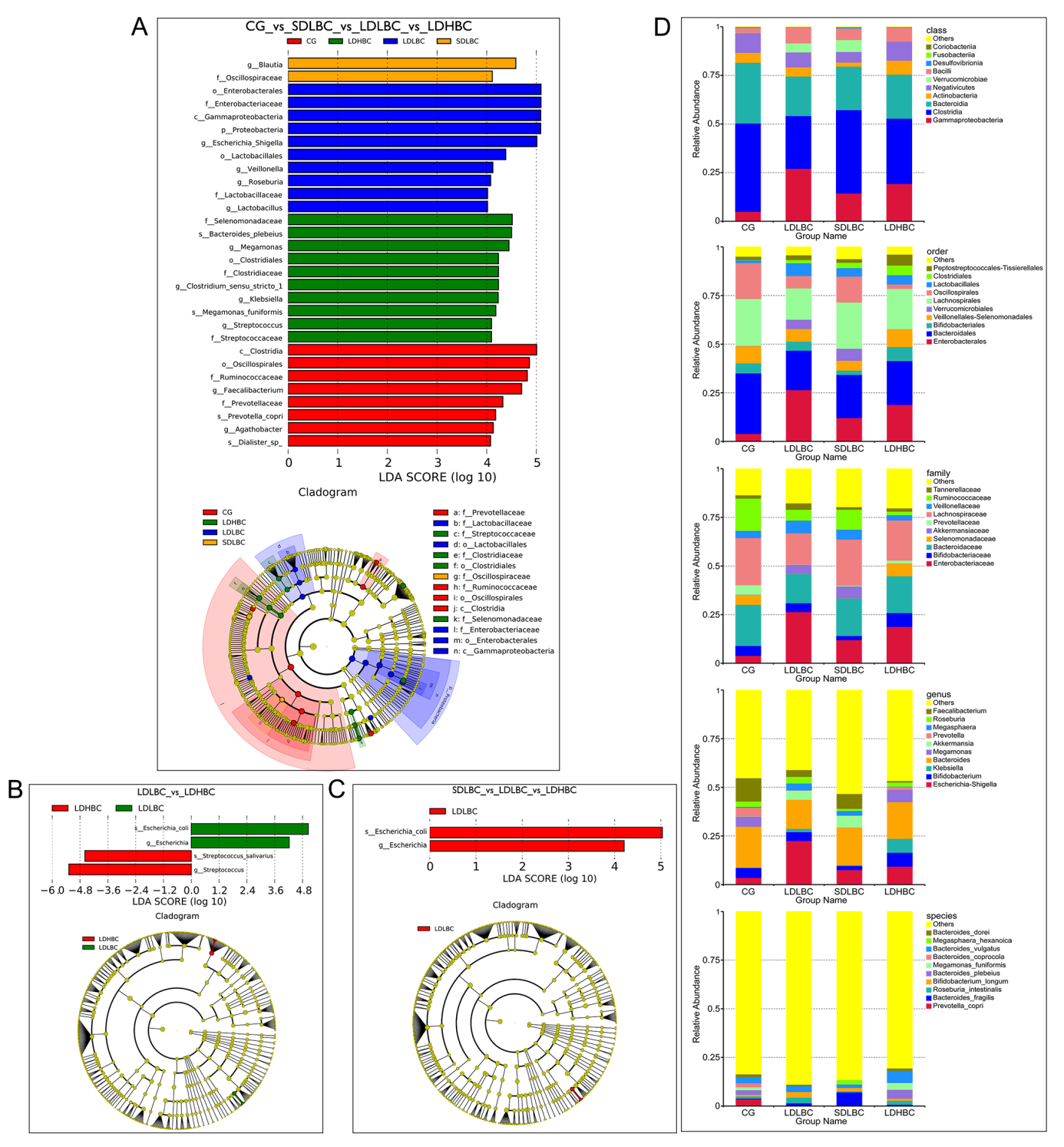
**

**Figure S2.** **Species differences between groups annotated by 16S rDNA amplicon sequencing**. **(A)** Characteristic bacteria were identified among the four groups via LEfSe analysis on the basis of 16S rDNA amplicon sequencing data(LDA > 4). A clade diagram of different species is shown below. **(B&C)** Characteristic bacteria were identified between groups via LEfSe analysis on the basis of metagenomic sequence data(LDA > 4). **(D)** Bar plot showing the top 10 most abundant species at each taxonomic level (class, order, family, genus, and species), annotated on the basis of 16S rDNA amplicon sequencing.

**
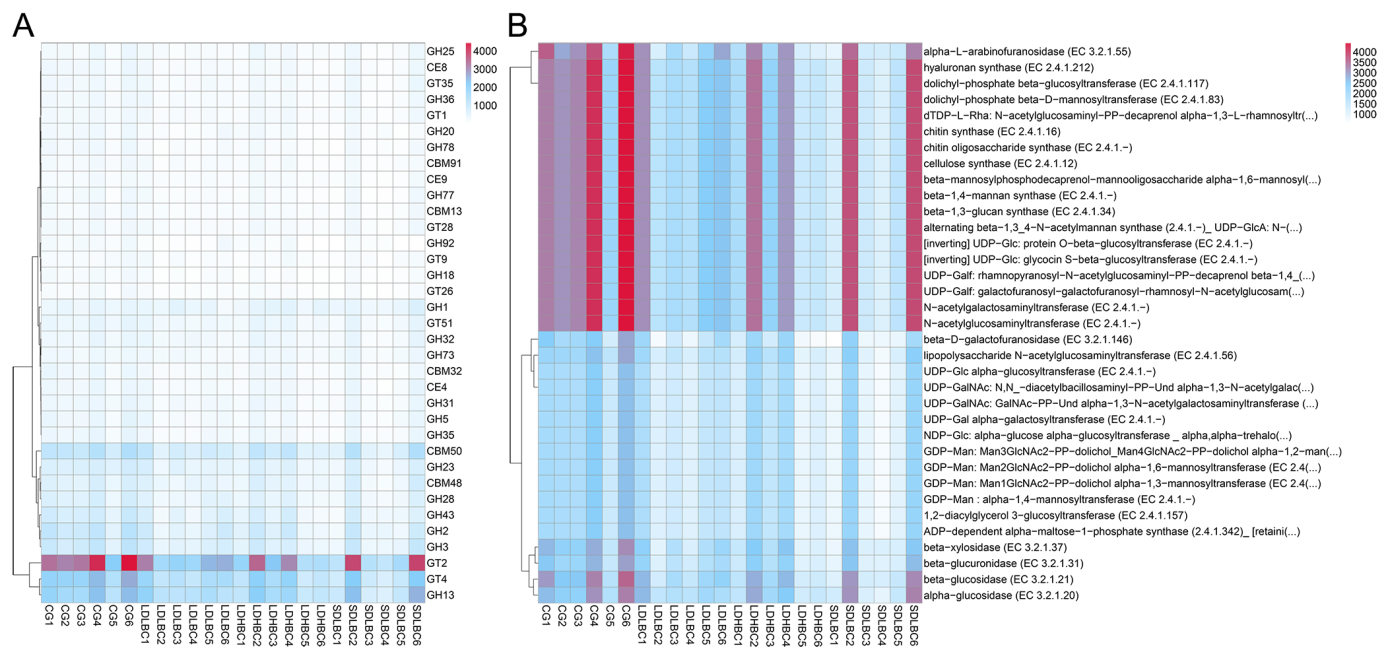
**

**Figure S3.** **Function Comments on the basis of the CAZy database**. **(A)** Heatmap analysis of gene counts annotated to level 2 genes (CAZy family). **(B)** Heatmap analysis of gene counts annotated to level 2 (EC number).

**
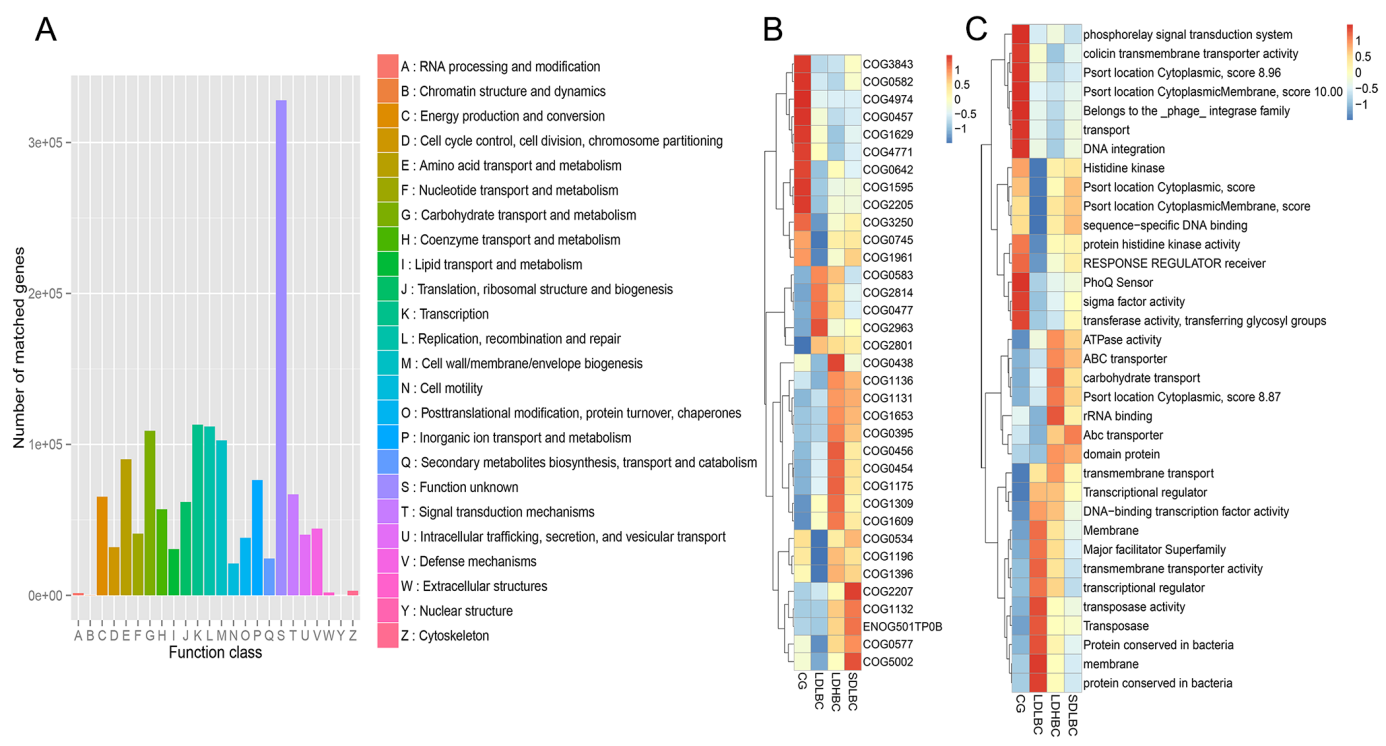
**

**Figure S4.** **Function Comments on the basis of the EggNOG database**. **(A)** Number of annotated genes in the EggNOG database (24 function classes). **(B)** Cluster analysis of functional relative abundance at the OG level (ortholog group ID). **(C)** Cluster analysis of functional relative abundance at level 2 (ortholog group description).

**
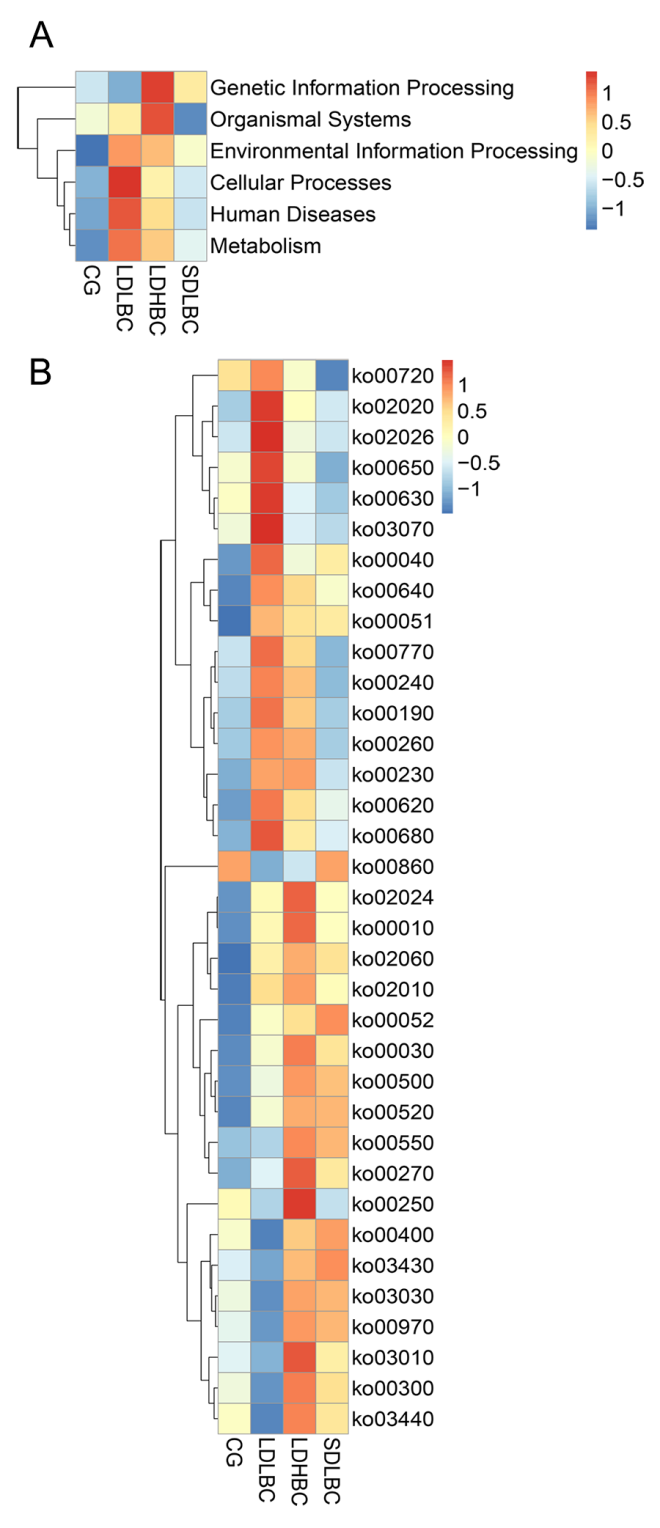
**

**Figure S5.** **Relative abundance clustering heatmap of the KEGG database**. **(A)** Relative abundance clustering heatmap of functions at level 1. **(B)** Relative abundance clustering heatmap of functions at level 3.

**
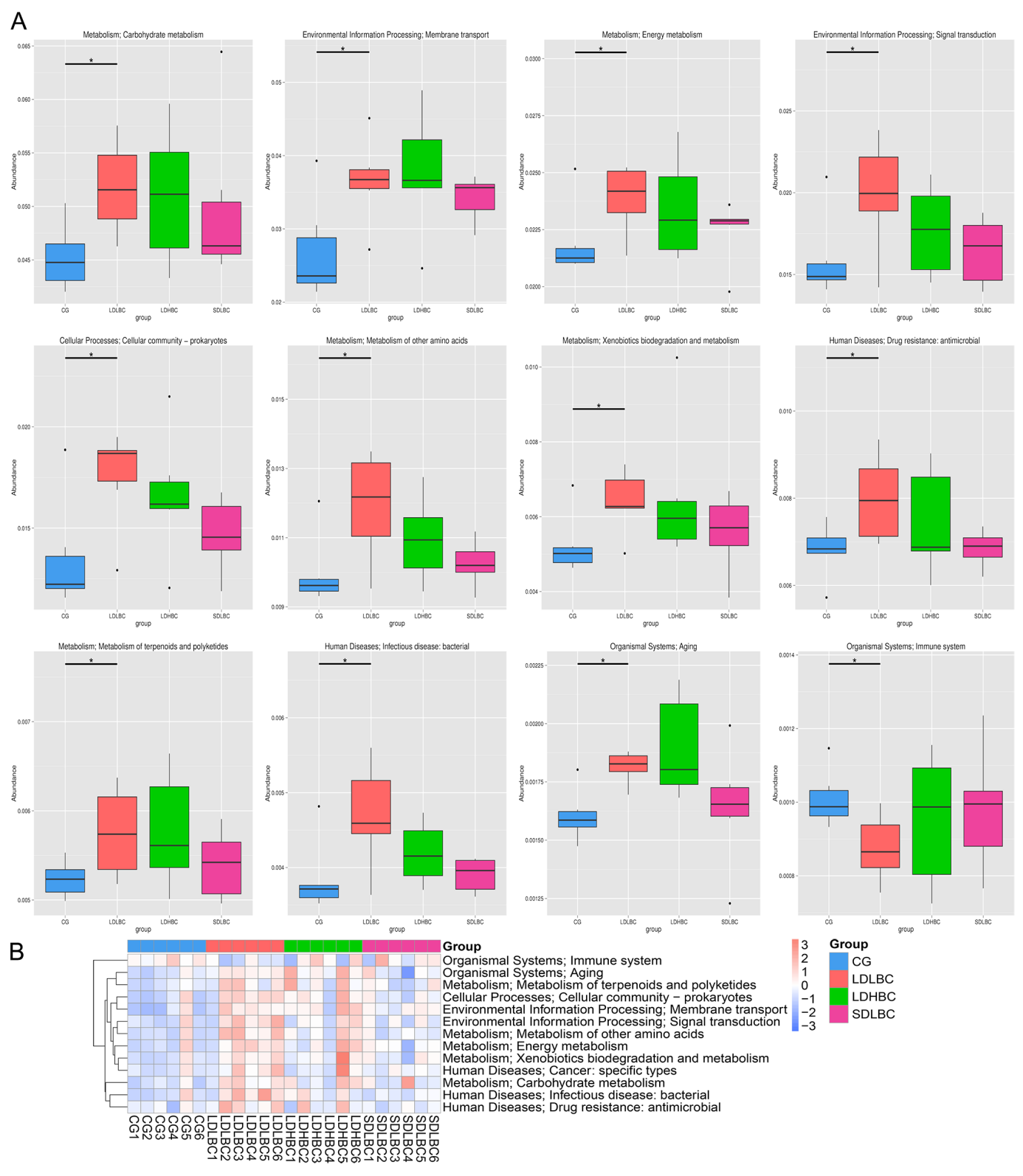
**

**Figure S6. Functional differences between groups**. **(A)** Metastat analysis of level 2 functional differences between groups (CG vs LDLBC). (The vertical axis represents the absolute abundance of the corresponding function, * p<0.05). **(B)** Abundance clustering heatmap of significantly different functions at level 2.

**
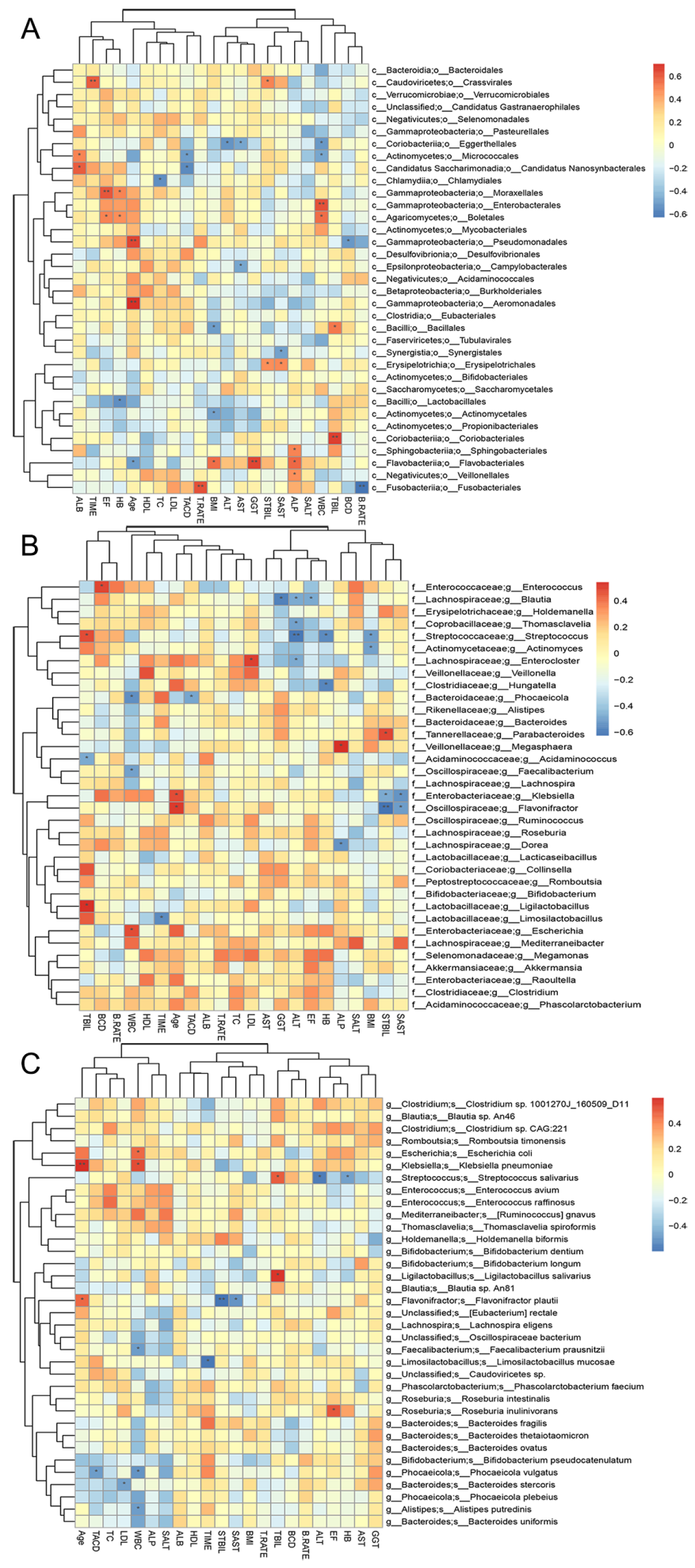
**

**Figure S7.** **Spearman correlation analysis of clinical characteristics and flora based on metagenomic sequence data.** (**(A)** Order, **(B)** genus, and **(C)** species). (The horizontal axis represents clinical characteristics, and the vertical axis represents the bacterial community; Spearman's r (between -1 & 1) in the heatmap center indicates correlation: r<0 for negative, r>0 for positive). (* p<0.05, ** p<0.01)
