# Supplementary material for: Influence of the gut microbiota on the pharmacokinetics of tacrolimus in liver transplant recipients: insights from microbiome analysis
Source: Front Microbiol. 2025 Sep 22;16:1616985. doi: 10.3389/fmicb.2025.1616985 (PMC12498155; doi:10.3389/fmicb.2025.1616985)
Supplement: Supplementary file 1 [file Supplementary_file_1.zip › Supplementary Materials and Methods.DOCX]

**Supplementary materials and methods**

1. Inclusion criteria and sample collection

We provided all study subjects with clean stool collection kits and instructed them to contact us immediately after collecting the samples. We would obtain stool specimens within half an hour and store them in cryotubes. The specimens were first rapidly cooled in liquid nitrogen for 15 minutes and then transferred to a -80°C freezer for storage until DNA extraction was performed. During these visits, we recorded the patient’s oral tacrolimus dosage (hereafter called TACD, mg/d) and tacrolimus blood drug concentrations (hereafter called BCD, ng/ml). Patients who had undergone liver transplantation for at least two months were included in the study to minimize the significant impact of antibiotics used during hospitalization on the gut microbiota and to avoid potential biases caused by short-term postoperative gastrointestinal dysfunction or irregular dietary habits. Exclusion criteria included patients not taking oral tacrolimus, those who had their tacrolimus dosage adjusted within the past three weeks, individuals presenting with symptoms such as constipation, hematochezia, diarrhea, indigestion, or other gastrointestinal disorders, as well as patients with complications requiring antibiotic treatment or hospitalization. Participants in the CG group had not used antibiotics in the past three months.

1. Treatment characteristics of liver transplantation patients

All liver transplant patients began immunosuppressive combination therapy with tacrolimus, glucocorticoids, and mycophenolic acids (or sirolimus) within one week after surgery. When the patient was allowed to eat, routine oral probiotics (Live Combined Bifidobacterium and Lactobacillus Tablets: *Bifidobacterium Longiformis*, *Lactobacillus Bulgaricus*, *Streptococcus thermophilus*; and Clostridium Butyricum: live *Clostridium butyricum*) were given until discharge and antibiotic treatment was given during intraoperative and postoperative, which was discontinued before discharge.

1. 16S rDNA amplicon sequencing

We first sequenced the DNA extracted from stool specimens via 16S rDNA amplicon sequencing (16SV34) to obtain amplicon sequence variants (ASVs). We used bioinformatics tools, including the Silva 138.1 database and QIIME2 software (version QIIME2-202202), to annotate and analyze the ASVs gene sequences of the microbial samples, thereby gaining insights into the microbial composition and diversity. Per the manufacturer’s instructions, we extracted genomic DNA from stool specimens via the Magnetic Soil and Stook DNA Kit (TianGen, China, Catalog #: DP712). 16SV34 primer sequences are 341F (CCTAYGGGRBGCASCAG) and 806R (GGACTACNNGGGTATCTAAT). To all the PCR mixtures, we added 15 µL Phusion® High-Fidelity PCR Master Mix (New England Biolabs), 0.2 µM primers, and a 10ng genomic DNA template. The PCR mixtures were then subjected to an initial denaturation at 98℃ for one minute, followed by 30 cycles of denaturation at 98℃ (10 seconds), annealing at 50℃ (30 seconds), and extension at 72℃ (30 seconds), with a final extension at 72℃ for five minutes. Then, we purified the qualified PCR products using magnetic beads and quantified them using the enzyme standard method. According to the concentration of the PCR products, we thoroughly mixed them in equal amounts and checked them by electrophoresis on a 2% agarose gel. We recovered the target bands using the Universal DNA Purification Kit (TianGenn, China, Catalog #: DP214). Library construction was performed using the NEB Next® Ultra™ II FS DNA PCR-free Library Prep Kit (New England Biolabs, USA, Catalog #: E7430L). After the detection library was qualified, NovaSeq6000 was used for PE250 computer sequencing. Sequencing data were processed (split, merged, quality-filtered, and chimera-removed) to obtain effective tags (effective tags). The Effective Tags are denoised by the DADA2 module in QIIME2(Version Qiime2-202202) software to obtain the final ASVs and feature list. Species annotation against the Silva 138.1 database via the classify-sklearn algorithm of QIIME2 software.

1. Bioinformatics analysis

We performed alpha and beta diversity analyses to assess the species diversity and composition of the samples. Alpha diversity indices (observed ASVs, shannon, simpson, chao1, goods coverage, dominance, and pielou’s evenness) were calculated via QIIME2 and visualized via R (v4.0.3). Beta diversity analysis (weighted and unweighted UniFrac distances) was performed via QIIME2. The species relative abundance results (barplot and phylogenetic tree) were generated via R software, while the heatmap and Venn diagram were produced via Perl (v5.26.2). The heatmap displays the Z values of the microbial taxa in each group, calculated as the difference between the group’s relative abundance at a given taxonomic level and the mean relative abundance of all groups at that level, divided by the standard deviation of the relative abundance across all groups. Statistical analyses (anosim, t test, UPGMA clustering, and LEfSe) revealed significant differences in community structure.

1. Metagenomics sequencing

The DNA extraction and sample detection methods were identical to those employed for 16S rDNA amplicon sequencing. We took 1 μg of sample genomic DNA and randomly fragmented it into approximately 350 bp fragments via a Covaris ultrasonic fragmentation instrument. After library construction, we performed preliminary quantification via Qubit 2.0. Subsequently, we used Agilent 2100 to check the insert size of the library. After the library passed the inspection, we pooled different libraries and performed Illumina PE150 sequencing. We used Readfq to remove reads containing more than a certain percentage (default value 40bp) of low-quality bases (mass value ≤ 38) from raw data obtained by Illumina HiSeq sequencing platform; remove reads with a certain proportion of N-bases (10bp by default); remove reads whose overlap with the adapter exceeds a certain threshold (15bp by default). We utilized Bowtie2 (v2.2.4) software to filter out reads that potentially originated from the host. We performed assembly analysis via MEGAHIT (v1.0.4) software on the clean data obtained after preprocessing. Starting from the Scaftigs (≥ 500 bp), we performed the open reading frame (ORF) prediction via MetaGeneMark (v2.10). Subsequently, we deduplicated the ORF prediction results via CD-HIT (v4.5.8) software to obtain the initial gene catalog (genes). We used a default identity of 95% and coverage of 90% for clustering and selected the most extended sequence as the representative sequence. We mapped the clean data from each sample to the initial gene catalog via Bowtie2 (v2.2.4). Genes with > 2 reads were filtered out to obtain the final gene catalog (Unigenes) for subsequent analysis. The abundance information of each gene in each sample was calculated based on the number of reads mapped and the gene length using the following formula:

$$G_{k}=\frac{r_{k}}{L_{k}}\cdot\frac{1}{\sum_{i=1}^{n} \frac{r_{i}}{L_{i}}}$$

(Note: r is the number of reads, and L is the length of the gene)

Based on the abundance of information on each gene in each sample in the gene catalog, basic information statistics, core-pan gene analysis, correlation analysis between samples, and venn diagram analysis of gene number were carried out. Library preparation and sequencing were performed at Novogene Co. Ltd.

1. Species, function, and antibiotic-resistance gene annotation

We used the DIAMOND software (https://github.com/bbuchfink/diamond/) to align the Unigenes with bacterial, fungal, archaeal, and viral sequences extracted from the NCBI NR database (https://www.ncbi.nlm.nih.gov/). We also aligned the Unigenes with functional databases, including the kyoto encyclopedia of genes and genomes database (KEGG, http://www.kegg.jp/kegg/), the evolutionary genealogy of genes: non-supervised orthologous groups database (eggNOG, http://eggnog6.embl.de/#/app/home), and the carbohydrate-active enzymes database (CAZy, http://www.cazy.org/)(Drula et al., 2022). We used the resistance gene identifier (RGI v6.0.2) software provided by the comprehensive antibiotic resistance database (v3.2.6) (CARD, https://card.mcmaster.ca/) to align the Unigenes with the CARD database. Based on the RGI alignment results and the abundance information of the Unigenes, we calculated the relative abundances of various antibiotic resistance ontology (ARO) terms. Finally, we performed relevant analyses based on the ARO abundances.

**References**

Drula, E., Garron, M.L., Dogan, S., Lombard, V., Henrissat, B., and Terrapon, N. (2022). The carbohydrate-active enzyme database: functions and literature. *Nucleic Acids Res* 50(D1)**,** D571-D577. doi: 10.1093/nar/gkab1045.
