# Supplementary material for: Influence of the gut microbiota on the pharmacokinetics of tacrolimus in liver transplant recipients: insights from microbiome analysis
Source: Front Microbiol. 2025 Sep 22;16:1616985. doi: 10.3389/fmicb.2025.1616985 (PMC12498155; doi:10.3389/fmicb.2025.1616985)

**Supplementary Materials pathwaymaps**

1. map00680 Methane metabolism

2. map00100 Steroid biosynthesis

3. map00140 Steroid hormone biosynthesis

4. map05130 Pathogenic Escherichia coli infection

5. map05225 Hepatocellular carcinoma

6. map04625 C-type lectin receptor signaling pathway; map04662 B cell receptor signaling pathway; map04650 Natural killer cell mediated cytotoxicity; map04062 Chemokine signaling pathway


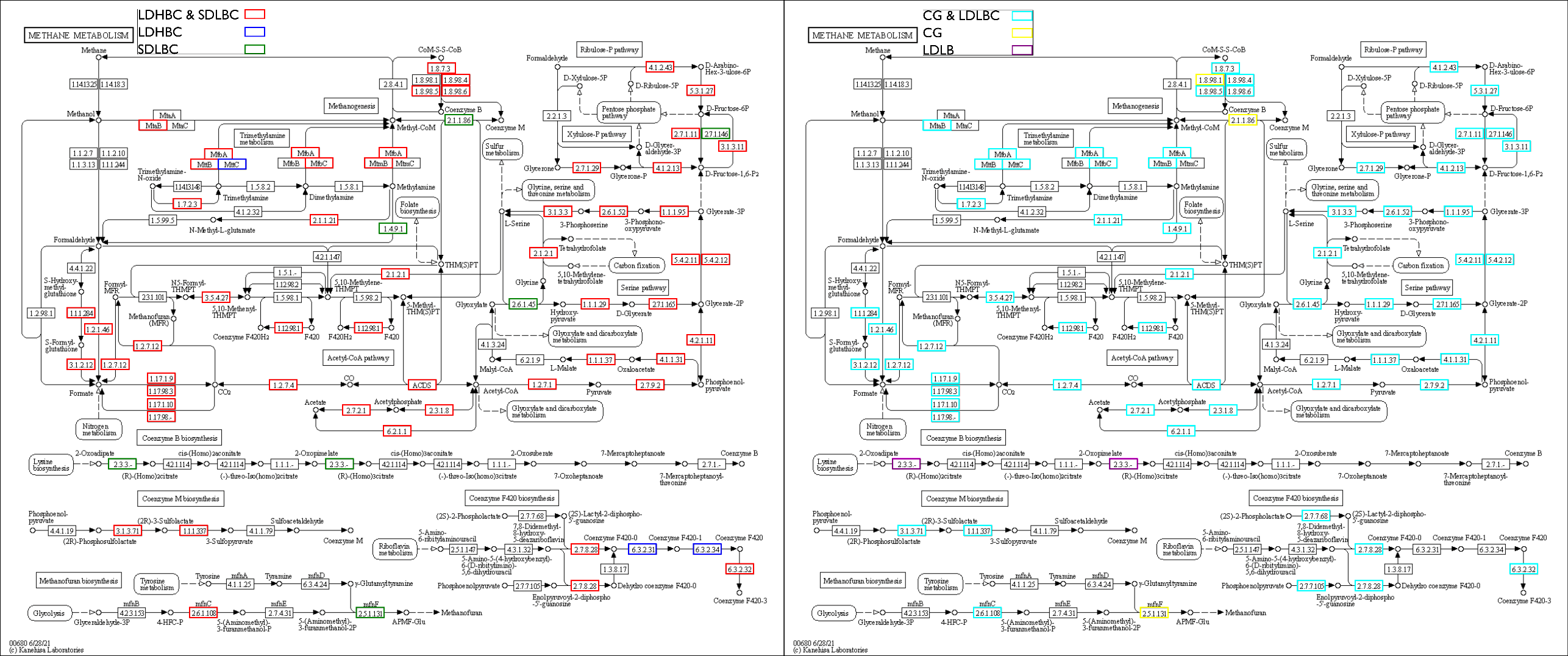


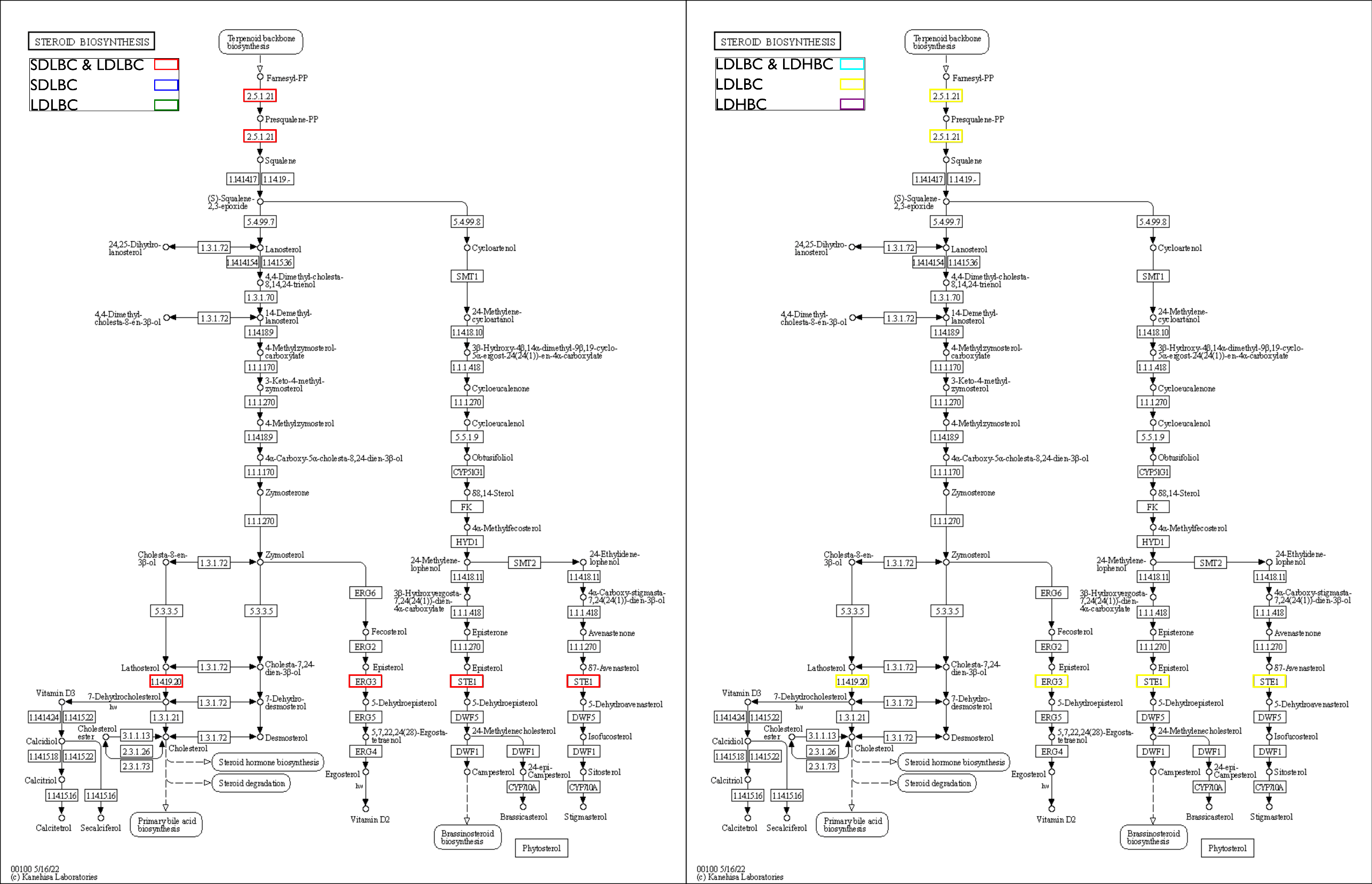

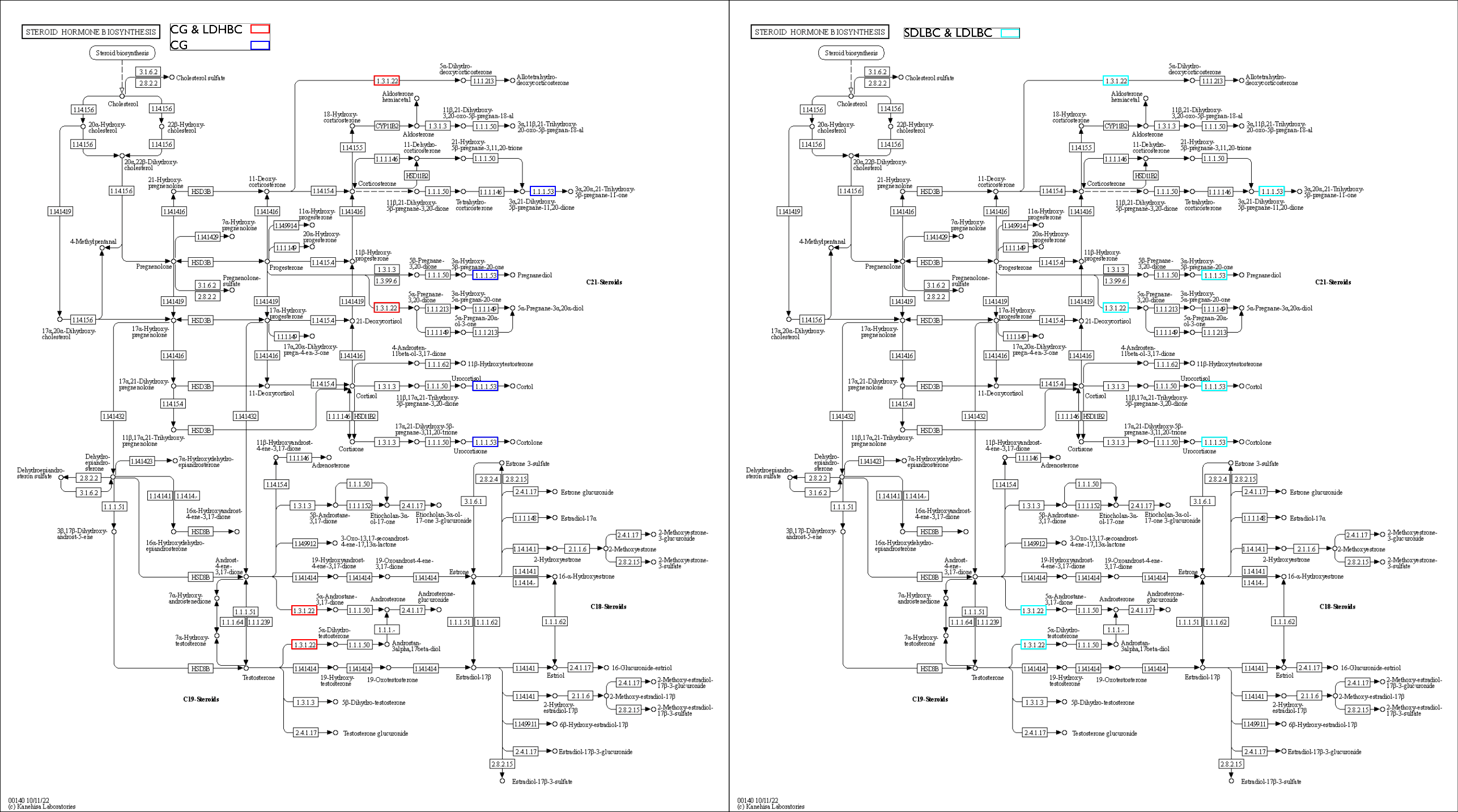


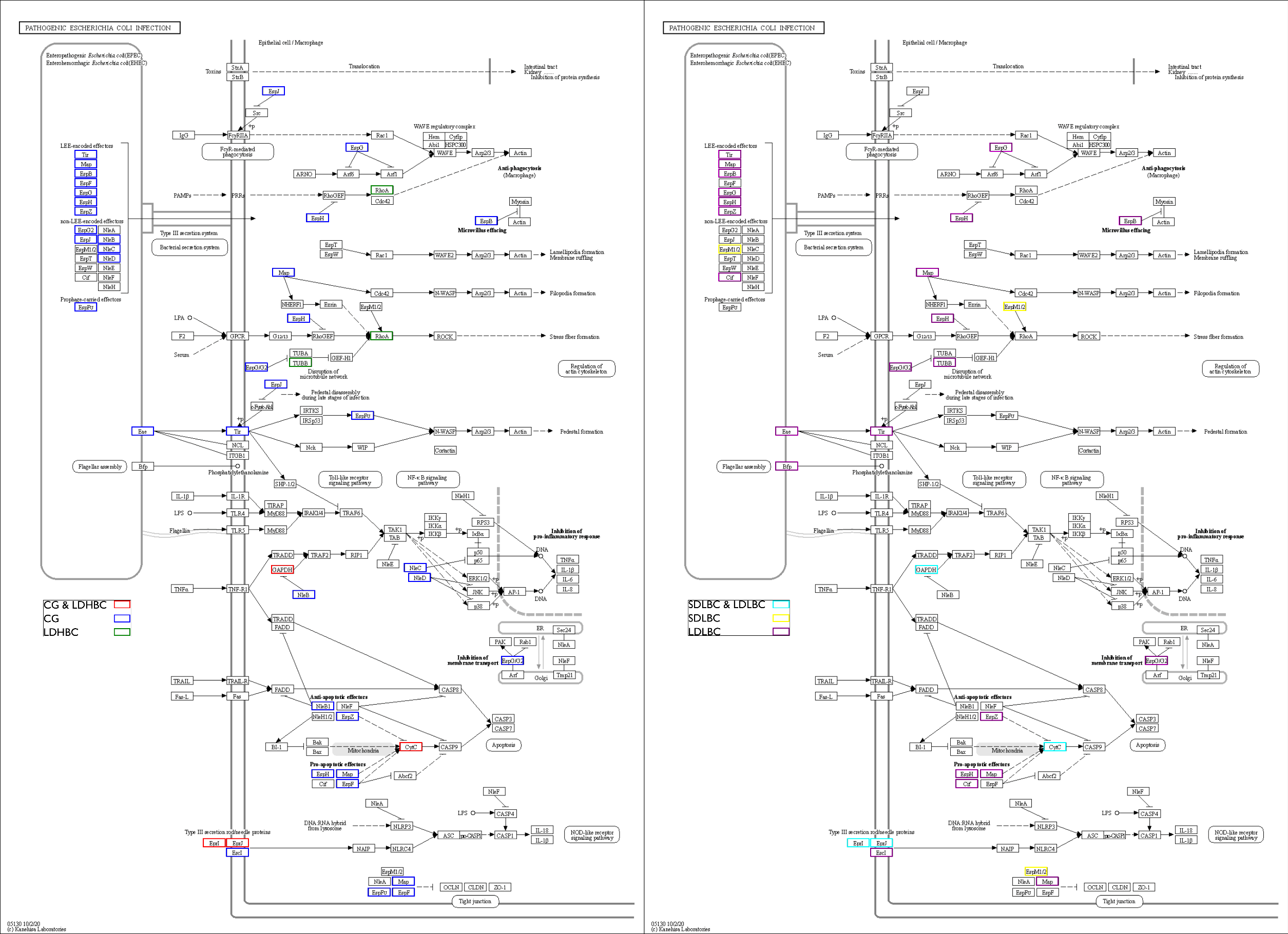


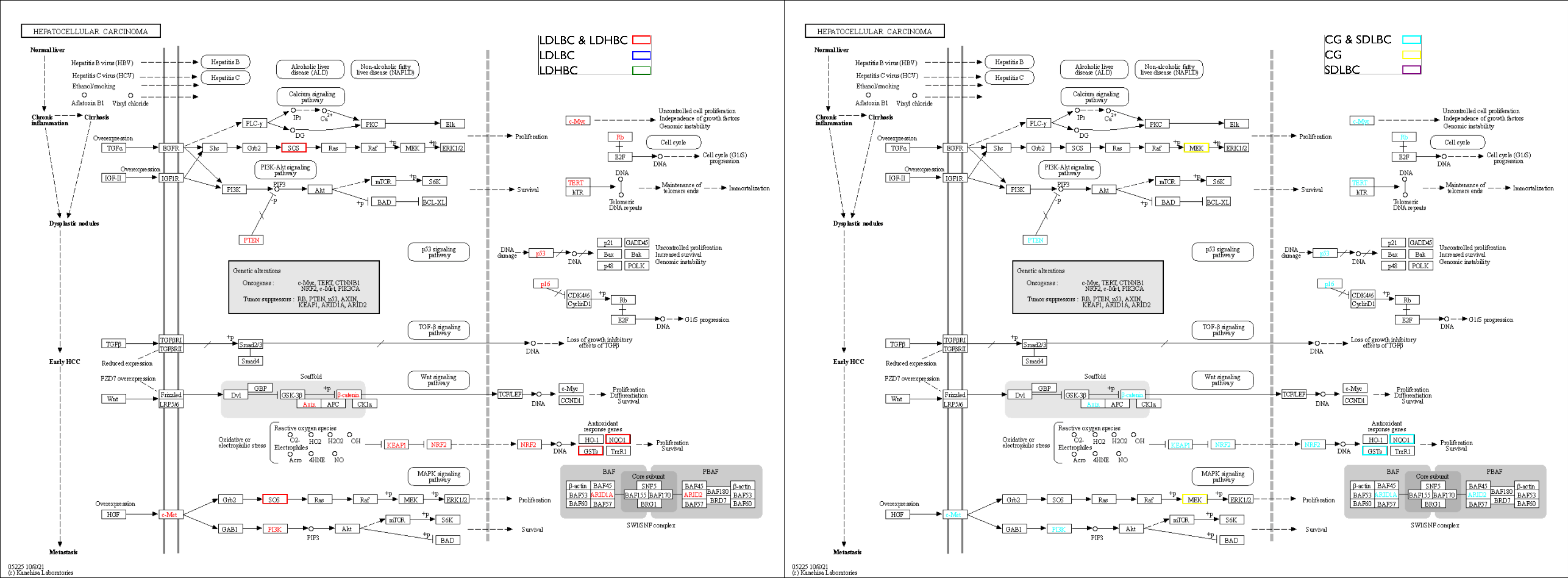


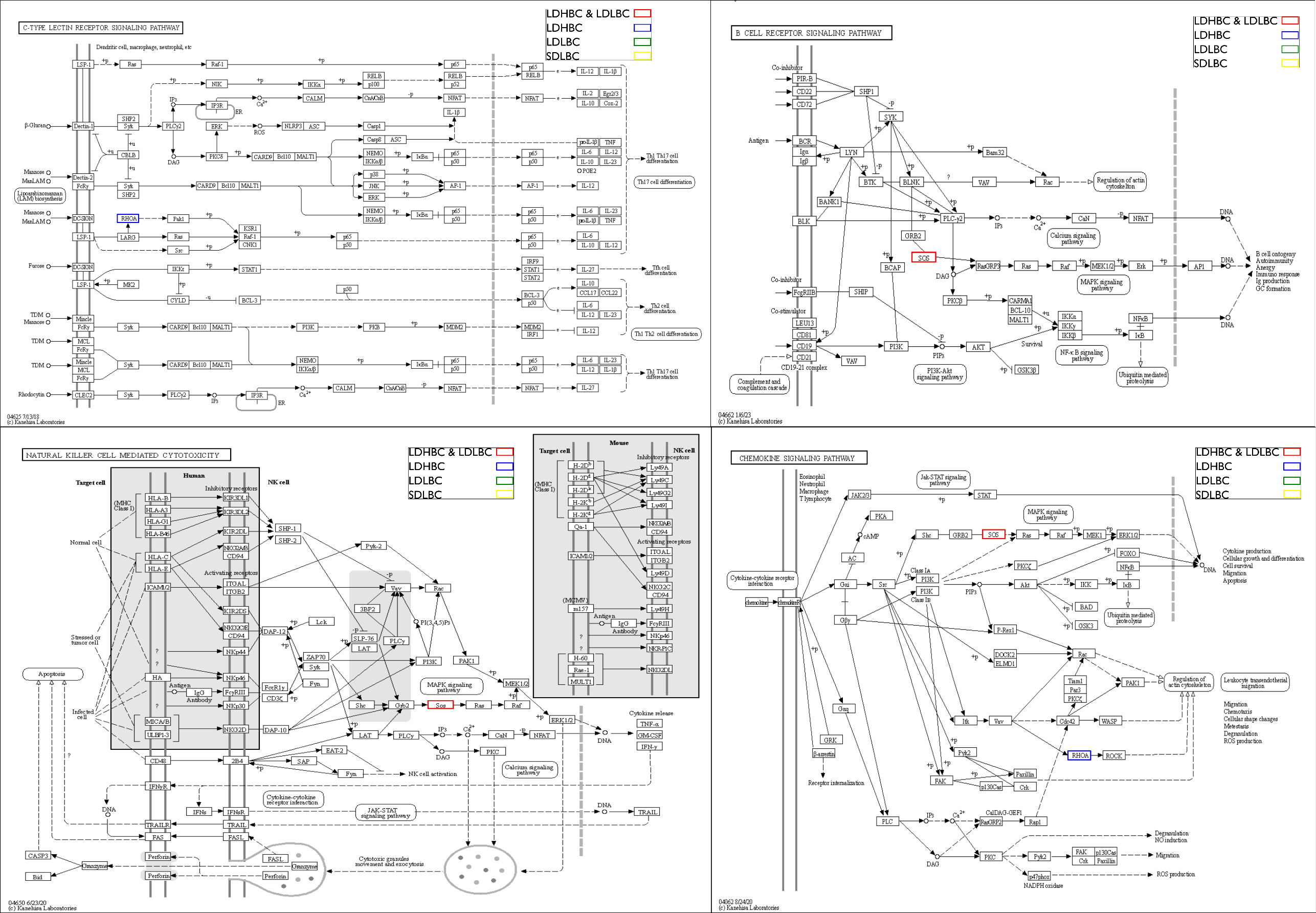

Supplement: Supplementary file 1 [file Supplementary_file_1.zip › Supplementary Pathwaymaps.DOCX]
